# Heterotopic Caval Valve Implantation for Severe Tricuspid Regurgitation: A Systematic Review and Recommendations for Implantation and Futility

**DOI:** 10.31083/RCM51423

**Published:** 2026-07-21

**Authors:** Diletta Corrado, Antonio Nenna, Mohamad Jawabra, Benedetto Ferraresi, Carmelo Dominici, Giovanni Casali, Filippo Toriello, Stefano Carugo, Massimo Chello, Mario Lusini

**Affiliations:** ^1^Cardiac Surgery, Fondazione Policlinico Universitario Campus Bio-Medico, 00128 Rome, Italy; ^2^Department of Cardio-Thoracic-Vascular Diseases, Foundation IRCCS Ca’ Granda Ospedale Maggiore Policlinico, 20122 Milan, Italy; ^3^Cardiac Surgery, Azienda Ospedaliero Universitaria Maggiore della Carità di Novara, 28100 Novara, Italy; ^4^Department of Clinical Sciences and Community Health, Università degli Studi di Milano, 20122 Milan, Italy

**Keywords:** tricuspid regurgitation, caval valve implantation, heterotopic valve, TricValve, TRICENTO, transcatheter therapy

## Abstract

**Background:**

Heterotopic transcatheter caval valve implantation (CAVI) has emerged as a palliative yet promising therapeutic strategy for treating severe tricuspid regurgitation (TR), which aims to reduce systemic venous congestion by implanting bioprosthetic valves in the venae cavae rather than in the native tricuspid annulus. However, despite encouraging procedural success, clinical improvement and survival remain highly variable.

**Methods:**

A systematic review of published registries and case series was performed, focusing on the outcomes, procedural success, mortality, and safety of CAVI. Data were extracted from multicenter registries and observational series describing the use of transcatheter stented bioprosthesis or bioprosthesis alone.

**Results:**

Across published cohorts and registries, procedural success rates ranged from 90% to 100%, with 30-day mortality ranging from 5% to 25%. The TRICUS EURO study, which included 35 patients across 12 European centers, demonstrated significant improvements in New York Heart Association (NYHA) functional class and Kansas City Cardiomyopathy Questionnaire (KCCQ) scores at 6 months, with minimal device-related complications and adverse events. The ongoing TRICAV-II pivotal trial (NCT06458907) and EuroTR registry (NCT06307262) are expected to provide larger real-world datasets. Smaller case series and individual reports, typically involving ≤10 patients, confirmed feasibility and symptomatic benefits, particularly reductions in peripheral edema and ascites. However, long-term outcome data remain limited. This study highlights right ventricle (RV) dysfunction, severe pulmonary hypertension, advanced end-organ failure, and clinical frailty as major determinants of unfavorable outcomes.

**Conclusions:**

Heterotopic CAVI represents a viable alternative for high-risk patients with severe TR, offering symptomatic improvement and reduced venous congestion when conventional surgery or orthotopic repair is not feasible. However, current evidence is derived primarily from non-randomized studies and limited registries. Ongoing prospective registries and pivotal trials are crucial for defining patient selection, procedural optimization, and long-term survival benefit. Early intervention and multidisciplinary patient selection appear crucial for avoiding futile procedures and identifying patients who may derive true symptomatic and prognostic benefit from CAVI.

## 1. Introduction

Tricuspid regurgitation (TR) is the third most common valvular heart disease after mitral regurgitation and aortic stenosis. The prevalence of TR increases with age and is more frequently encountered in women and in patients with atrial fibrillation and heart failure (HF) [1]. Moreover, TR is often associated with left-sided valvular disease, but may also present as isolated TR, which is associated with increased mortality even in the absence of other cardiopulmonary comorbidities. Severe TR is associated with an increased risk of HF and death, independent of pulmonary pressure, ventricular function, and comorbidities [2]. The etiology of TR is most often secondary (90%), resulting from atrial or ventricular dilatation that leads to annular dilatation. TR is increasingly recognized as a marker of poor prognosis and advanced right HF. Traditional surgical repair or replacement carries high operative mortality, particularly in patients with prior left-sided valve surgery, right ventricular dysfunction, or end-organ impairment [3]. Several transcatheter procedures have emerged for patients with severe isolated TR who are at a high or prohibitive surgical risk. However, many patients remain unsuitable for transcatheter procedures, such as transcatheter tricuspid edge-to-edge repair (T-TEER), annuloplasty, or orthotopic valve replacement, due to complex anatomy or marked annular dilatation [4]. In this context, the development of transcatheter tricuspid interventions has expanded therapeutic options for patients with severe to torrential TR who have no other options for alleviating congestive symptoms. Among these procedures, caval valve implantation (CAVI) has emerged as a heterotopic, minimally invasive alternative designed to reduce venous congestion by implanting bioprosthetic valves in the superior and inferior vena cava (IVC). Early experience has demonstrated technical feasibility and short-term symptom relief from right-sided congestion, including peripheral edema, ascites, and hepatic congestion, without directly intervening on a structurally compromised right ventricle or annulus [5]. Early single-arm studies and multicenter registries, including TRICUS, TRICUS EURO, and TRICAVAL, have shown that CAVI is technically feasible, associated with high procedural success, and capable of improving New York Heart Association (NYHA) functional class and quality of life. However, long-term survival remains dependent on right ventricular function and comorbidities. Consequently, CAVI represents a valuable therapeutic option for patients considered inoperable or at extreme surgical risk. Nevertheless, the long-term survival benefit of CAVI remains uncertain, and many patients continue to die despite procedural success. Therefore, understanding when CAVI becomes clinically futile, or when mortality remains high despite treatment, is essential for appropriate patient selection and ethical decision-making. This systematic review aims to define criteria for avoiding CAVI in patients unlikely to benefit from the procedure by examining data from case series and registries.

## 2. Materials and Methods

This systematic review was conducted through a comprehensive search of the PubMed, Scopus, and Embase databases, as well as ClinicalTrials.gov and other registries. The following search terms were used: “caval valve implantation”, “heterotopic valve”, “tricuspid valve”, “tricuspid regurgitation”, “TricValve”, “TRICENTO”, and “bicaval valve”. Each source was last consulted on 1st November 2025. We included registries, case series, and clinical trials. Eligible articles were identified based on the following criteria: (1) registries, case series, and randomized clinical trials; (2) studies of adult patients (>18 years) with tricuspid valve regurgitation treated with CAVI; (3) studies reporting procedural success, outcomes, and mortality to evaluate the impact and futility of CAVI. We excluded editorials, conference reports, and abstracts. Articles concerning transcatheter procedures other than CAVI were also excluded. No restrictions were placed on publication date or language. In addition, both backward and forward citation chain analyses were performed for all included studies to identify further relevant publications. Eligibility criteria were defined according to the PICO framework and are summarized in** Supplementary Tables 1 and 2**. The population included adult patients with severe or greater TR who remained symptomatic despite optimized medical therapy (OMT), were unsuitable for surgery and/or at high surgical risk, and were also unsuitable for other transcatheter procedures (*e*.*g*., T-TEER). The intervention of interest was transcatheter CAVI. Studies reporting a comparator group (*e*.*g*., medical therapy) were included when available. Otherwise, data on symptom improvement, improvement in liver and renal congestion, reduced diuretic dose, and decreased peripheral edema were collected. Outcomes of interest included mortality, procedural success, HF readmission, and procedure-related complications. Two authors independently reviewed the complete list of articles obtained from the systematic search. After duplicates were removed, titles and abstracts were screened. Potentially eligible articles meeting the eligibility criteria underwent full-text review, and reasons for exclusion were recorded. Data were collected by one reviewer, who completed separate tables for registries and case series, followed by a cross-check between the structured tables and the selected studies, also performed by the same reviewer, to minimize data gaps. All structured tables and collected data were independently checked by another reviewer to minimize the risk of bias. We tabulated study characteristics, including intervention, population, comparator, and outcomes. Studies were then assigned to each synthesis based on the availability of relevant outcomes (*e*.*g*., 30-day mortality, procedural success, 1-year survival). For each outcome, data are reported as presented in the original studies. Dichotomous outcomes (*e*.*g*., mortality, procedural success, rehospitalization) and continuous outcomes (*e*.*g*., NYHA class, quality-of-life scores) are presented as percentages. No quantitative synthesis or pooled effect measures were calculated due to heterogeneity among the included studies.

We extracted the following data from the studies:

- Study characteristics: author, publication year, sample size, and inclusion and exclusion criteria;

- Procedural characteristics: type of implanted valve (Edwards SAPIEN XT or 3, TRICENTO, TricValve), procedural success, and complications;

- Outcomes: symptom improvement, improvement in liver and renal congestion, hospitalization for HF, and mortality.

Data were extracted and are summarized as reported in the original publications. Missing summary statistics were not imputed. When outcomes were reported in different formats, data were standardized for descriptive purposes (*e*.*g*., by converting absolute numbers to percentages). Formal statistical methods to assess heterogeneity, such as subgroup analysis or meta-regression, were not applied. Heterogeneity among studies was explored descriptively by examining differences in study design, patient populations, intervention characteristics, and outcome definitions. Owing to substantial heterogeneity in study design, outcome definitions, and follow-up duration, a quantitative, statistical meta-analysis and sensitivity analysis were not performed. The robustness of the results was assessed qualitatively, and trends in outcomes were consistent across study types, designs, and sample sizes. Results were synthesized using a narrative approach, grouping studies according to study design and intervention characteristics, and summarizing outcomes descriptively. The results are presented narratively. Where applicable, the direction of effect is indicated: most studies reported improved NYHA class, high procedural success rates, low 30-day mortality, and reduced rehospitalization. Clinical benefit was defined as improvement in symptoms and a low HF readmission rate. In contrast, futility was defined as a high mortality rate and/or a high rehospitalization rate for HF.

Included studies comprised prospective and retrospective observational cohort studies, multicenter registries, single-arm case series, and one randomized controlled trial. Methodological quality was assessed using the Joanna Briggs Institute (JBI) critical appraisal tools, selecting the appropriate checklist according to study design (cohort, case series, case report, or randomized controlled trial). Two reviewers independently evaluated each study, resolving discrepancies through discussion. Most registry-based and prospective cohort studies were judged to have a moderate risk of bias, primarily due to the associated observational design and lack of control groups. Case series and case reports were associated with a high risk of bias, primarily due to small sample size, descriptive design, and limited outcome reporting. The single randomized controlled trial showed a moderate risk of bias related to early termination and limited statistical power. In addition, each study was assigned a quality rating (high, moderate, or low) based on the associated JBI assessment, study design, sample size, completeness of follow-up, and generalizability. This rating provides an overall assessment of methodological robustness and informs the interpretation of the synthesized outcomes. The results of both the JBI assessment and quality rating are presented in **Supplementary Table 3**. Risk of bias due to missing results (reporting bias) was assessed qualitatively by evaluating the completeness of outcome reporting in the included studies. No formal statistical methods (*e*.*g*., funnel plots) were applied due to the small number of studies and associated heterogeneity. The certainty of the evidence was not formally assessed using methods such as GRADE due to the observational nature and heterogeneity of the included studies. Confidence in the reported outcomes was considered qualitatively based on study design, sample size, and completeness of outcome reporting. Overall, mortality and procedural success outcomes were supported by moderate-quality evidence, whereas NYHA and rehospitalization outcomes were supported by low- to moderate-quality evidence.

**Fig. 1.**
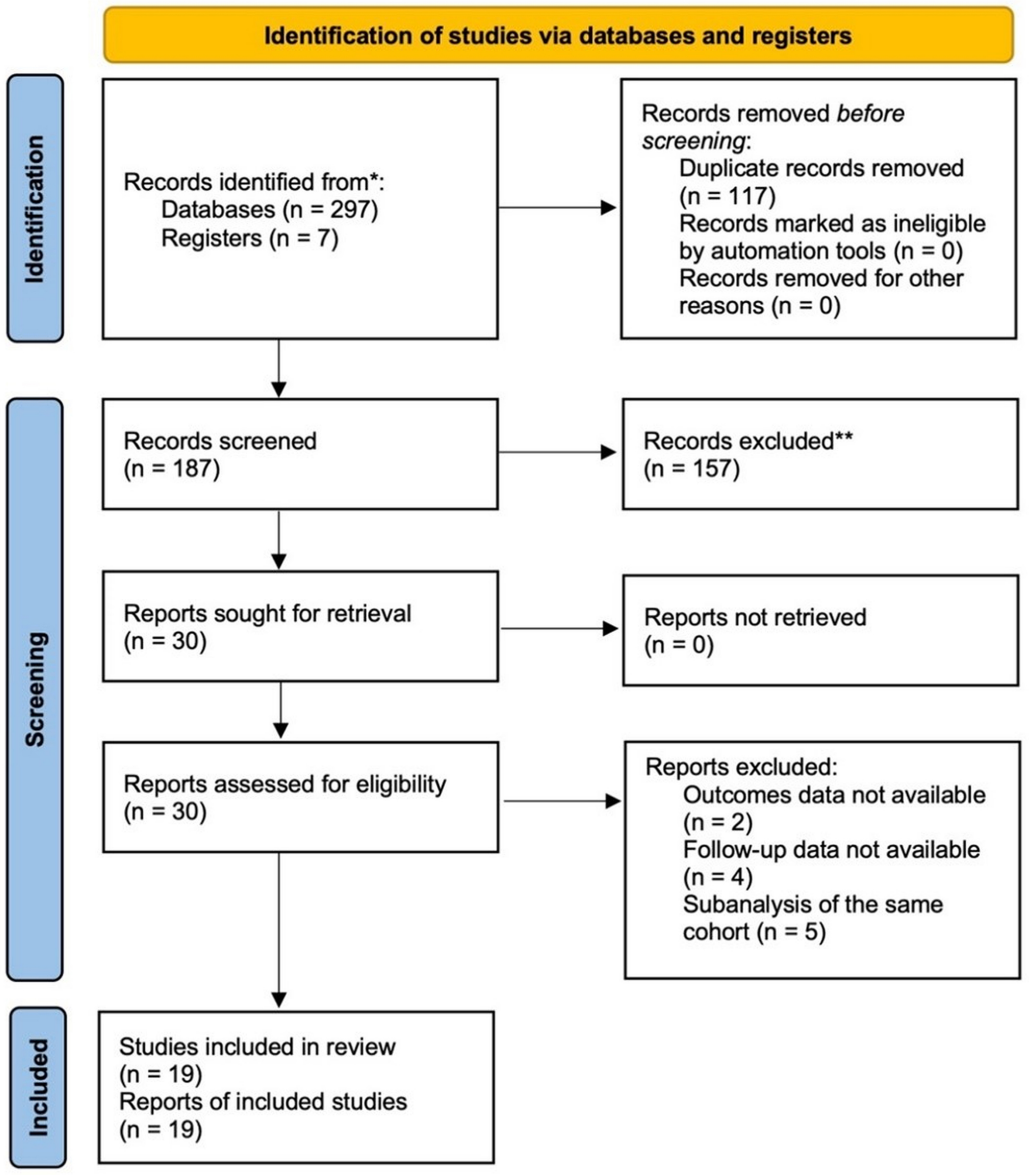
**PRISMA flow diagram**. PRISMA, Preferred Reporting Items for Systematic Reviews and Meta-Analyses. *Number of records identified from each database or register searched. **All records were screened and excluded without the use of any automation tools.

Overall, 304 studies were identified, including 297 from databases and 7 from registers. After exclusion according to prespecified criteria, 19 studies were included: 5 registries and 14 case series. Visual presentation included a Preferred Reporting Items for Systematic Reviews and Meta-Analyses (PRISMA) 2020 flow diagram for study selection (Fig. 1). Risk of bias was generally moderate in registry studies due to selection criteria and complete outcome reporting, and high in small case series. The only randomized controlled trial (RCT) included had a low-to-moderate risk of bias. Studies with no data on patient outcomes or follow-up were excluded owing to the absence of comparable information. Sub-analyses of the same cohort were excluded when the reported information was irrelevant to the aim of this study. This systematic review was not registered, and no protocol was prepared.

## 3. Results

The systematic search and study selection process is presented in the PRISMA flow diagram (Fig. 1). Of the reports assessed for eligibility, six were excluded for insufficient data on outcomes and follow-up, and five sub-analyses of the same cohort were also excluded. After these exclusions, 19 studies were included in the present review, comprising 5 registries and 14 case series. The methodological quality of the included studies is summarized in **Supplementary Table 3**. Overall, the quality of evidence was moderate to low, reflecting the predominance of early-phase studies, case series, and registry data.

Common limitations included small sample sizes, incomplete follow-up reporting, and lack of blinding in interventional studies. The only included randomized controlled trial [6] showed overall moderate quality, with some items rated as “unclear” due to incomplete reporting of allocation concealment and blinding procedures. Only two case series were published before 2020; during 2020 and 2021, seven additional case series were published. Moreover, three case reports were published during the last year. Unlike case series, fewer registries were available, with publications appearing only after 2020. Specifically, the first study registered on ClinicalTrials.gov was the randomized clinical trial, TRICAVAL, which started in 2015 and was completed in 2018, and included 28 patients (**Supplementary Tables 1 and 2**). As of 2020, four ongoing studies were available, with different estimated completion dates and larger planned sample sizes. However, among previously published studies, the first registry, the US Caval Valve Registry [7], included 24 patients who underwent implantation of the SAPIEN 3 valve (Edwards). This study demonstrated a 100% procedural success rate for valve implantation, with low procedural complication rates in 24 patients (bleeding 8.3%, major vascular complications 4.2%, and 1 procedure-related death). Meanwhile, 30-day mortality was 25%, in-hospital mortality was 20.8%, and overall mortality during a mean follow-up of 332 days was approximately 58.3%. The TRICUS EURO study [8], a CE-mark trial, evaluated the safety and efficacy of the TricValve system in 35 patients with severe symptomatic TR and high surgical risk over a 6-month follow-up period. Technical success was 97%, while procedural success was also higher (94%). There was one case of superior vena cava (SVC) prosthesis migration into the right atrium without embolization, which was managed conservatively and followed by early implantation of a permanent pacemaker (PMK). The 6-month mortality was 8.5%, with marked improvement in NYHA class, complete or partial reduction in fluid overload, and lower loop diuretic doses. However, the HF readmission rate was 20%, mostly due to right HF and largely related to declining renal function or respiratory infection. In addition, no significant changes were observed in echocardiographic parameters; patients showed a significant increase in NT-proBNP due to right atrial (RA) ventricularization, and no statistically significant improvement in the 6-minute walking test (6MWT). Wild et al. [9], in a multicenter registry evaluating early clinical experience with the TRICENTO bicaval valved stent graft, included 21 patients. Technical success was 100%, with no need for pacemaker implantation. There were two cases of paraprosthetic leakage, only one of which was severe, and vascular complications occurred in 19% of patients, of which 5% were major. Clinical signs of congestion resolved in most patients: seven patients (37%) still had peripheral edema compared with 95% at baseline, and one patient (5%) had persistent ascites compared with 29% at baseline. The 30-day all-cause mortality was 5%. Although these results are encouraging, the 1-year mortality rate was 24%, with 10% of deaths due to cardiovascular causes. The rehospitalization rate for right HF was 19%, and postprocedural echocardiography showed no changes from baseline in left or right ventricular dimensions or function. Another complication reported in the registry was stent fracture in 14% of patients, which did not worsen or alter valve function. Blasco-Turrión et al. [10] collected data from the TRICUS study, an early feasibility/first-in-human study including nine patients from Lithuania, and from the TRICUS EURO study, previously reported by Estévez-Loureiro et al. [8], to assess 1-year follow-up outcomes. The 6-month mortality was 6.8% and did not differ from 1-year mortality; the rate of cardiovascular death was 2.2%. Improvements in NYHA class and Kansas City Cardiomyopathy Questionnaire (KCCQ) scores were statistically relevant, and echocardiography showed a significant reduction in tricuspid annular diameter. Renal and liver function remained stable throughout follow-up; however, there was no evidence of hepatic vein backflow, NT-proBNP levels were lower, and diuretic doses progressively decreased. Nevertheless, the overall rate of HF rehospitalization was 29.5%, with most events occurring after 6 months.

The TricBicaval Registry [11] is the largest and most recent registry published. This study included 204 patients who underwent TricValve implantation, with outcomes collected within 1 year. Intraprocedural success was observed in 96.1% of patients, and 30-day clinical success was 83%, with five failures due to IVC prosthesis malposition and one case of SVC prosthesis migration, treated with a valve-in-valve procedure. Among major adverse events (19.1%), the rate of major cardiac complications was low (5.9%). Furthermore, cardiac complications occurred in 4.9% of patients, with cardiac tamponade and pacemaker implantation being the most frequent, each accounting for 33.3% of these events. At 1-year follow-up, 81.5% of patients were in NYHA class II or lower, 22.1% had persistent peripheral edema, and 4.9% had ascites, with a reduced rate of hepatic vein systolic flow reversal. However, the all-cause mortality rate was 18.6%, with an estimated 1-year Kaplan–Meier rate of 22.7%. In-hospital mortality was 8.3%, and cardiovascular causes accounted for a high proportion of in-hospital mortality (76.5%). In addition, the rate of unplanned hospitalization for HF was 26.9%, with an estimated Kaplan–Meier rate of 22.9%. Postprocedural echocardiography, at 1 year, showed no changes in right atrial size and a non-significant reduction in right ventricular basal and mid-ventricular end-diastolic diameter. Moreover, tricuspid annular plane systolic excursion (TAPSE) and right ventricular fractional area change (FAC) worsened slightly but significantly. In conclusion, the TricBicaval Registry identified the TRI-SCORE as an independent predictor of 1-year mortality (6.7% for scores ≤3 vs. 28.8% for scores ≥6). These data highlight that successful implantation does not guarantee meaningful survival, underscoring the need to define preprocedural indicators of futility.

Among case series, Lauten et al. [12] included 25 patients with severe symptomatic TR despite optimal OMT, who were unsuitable for surgery and were candidates for CAVI on compassionate clinical use, with high mortality scores (STS 14, EuroSCORE II 18.2). A total of 17 patients received Edwards SAPIEN XT or SAPIEN 3 valves, seven patients received TricValve, and one received Direct Flow. Overall procedural success was 92%, with two cases of valve migration: one case of immediate IVC valve migration requiring conversion to surgery; a second patient underwent surgery 10 days after implantation due to IVC valve migration. However, the implantation procedure was not associated with intraprocedural mortality. In this study, the 30-day mortality was 12%, the in-hospital mortality was 24%, and the 1-year mortality was 63%. The 30-day mortality and the in-hospital mortality were not related to cardiovascular causes (progressive multiorgan failure and septic complications), but the causes of 1-year mortality were not reported. The procedure was associated with successful resolution of hemodynamic backflow, a significant reduction in mean IVC and RA pressures, and symptomatic improvement in 84.2% of patients. Nevertheless, plasma NT-proBNP levels were markedly elevated at baseline and increased within 30 days after the procedure, likely due to right atrial ventricularization, as described by Estévez-Loureiro et al. [8] in the TRICUS EURO study.

Dreger et al. [6] randomized 28 patients to compare OMT with CAVI using Edwards SAPIEN XT implantation in the IVC, with follow-up at 1, 3, 6, and 12 months. No differences were observed in maximal oxygen uptake measured by spiroergometry. NYHA class improved by 1 class during the first 3 months in 46% of patients in the OMT group and 63% in the CAVI group, while the class remained unchanged in 38% of patients in the CAVI group and 46% in the OMT group. Despite this early improvement, no significant differences in NYHA class were found either during the first 3 months or over the entire follow-up period. Likewise, no differences were observed between groups in quality of life, 6MWT, NT-proBNP levels, or right heart function. All-cause mortality was higher in the CAVI group than in the OMT group (57% vs. 29%), whereas the rate of right HF was higher in the OMT group (21% vs. 4%). The rate of HF hospitalization was 29% in both groups. The TRICAVAL study, the only available randomized controlled trial on CAVI, stopped patient recruitment after four cases required conversion to open surgery due to valve dislocation and stent migration.

Aalaei-Andabili et al. [13] enrolled 6 patients in a CAVI study using the Edwards SAPIEN 3 valve in the IVC. Procedural success was 100%. After 30 days, patients showed improvement in symptoms (ascites, peripheral edema, and physical capacity), TR severity, and ejection fraction (EF), except for two patients. However, one patient (16.67%) with severe right ventricular dysfunction died after 3 months, and four patients (66.67%) were readmitted within 6 months for shortness of breath, lower-extremity edema, and ascites.

Di Mauro et al. [14] included 13 patients with severe or worse symptomatic isolated TR despite OMT, NYHA class III or higher, hepatic or peripheral congestion, high surgical risk, and suitability for TricValve implantation, with an EF of at least 35%. Procedural success was 100%. After 170 days, patients showed no signs of right HF, and NYHA class improved in 82% of patients (73% in NYHA class II and 9% in NYHA class I). Echocardiographic parameters also improved, including an increase in left ventricular EF (LVEF) and a reduction in right ventricular diameter and dysfunction. In addition, this study showed an improvement in the TR grade, likely related to right ventricular remodeling and reduced annular dimensions and tethering, along with lower serum NT-proBNP levels and reduced diuretic doses. During the follow-up period, the HF readmission rate was 0, and patients showed no signs of congestion, such as ascites or peripheral edema, and required fewer high-dose diuretics. Nevertheless, two patients (15.4%) with a TRI-SCORE of 65% died of renal or liver failure, 6 and 124 days after the procedure, and the survival rate at 170 days was 80.2%

Recently, O’Neill et al. [15] performed CAVI in 10 patients using the Edwards SAPIEN 3 valve in the IVC, together with an Edwards caval pre-stent to prevent valve displacement or embolization, as reported in earlier studies [6]. Procedural success was 90%, with one case failing because the IVC diameter could not be measured. No major bleeding or vascular complications were reported during the procedure. After 30 days, an improvement in the Kansas City Cardiomyopathy Questionnaire-Overall Summary (KCCQ-OS) score was observed, and after 6 months, the RA, SVC, and IVC dimensions had decreased, while cardiac output and cardiac index had increased. After 1 year, half of the patients were in NYHA class I and the other half were in NYHA class II, with improved right ventricle (RV) dysfunction in 1 of 8 patients and a decreased mean hepatic vein diameter. However, 1-year mortality was 20%, myocardial infarction occurred in 10% of patients at 1 year, and major bleeding occurred in 10%. Furthermore, 30% of patients were hospitalized for HF during the first year. On postoperative days 37 and 204, a pre-stent fracture was identified in 70% (n = 7) of patients, but this was not associated with adverse events and did not affect prosthetic heart valve function.

Lurz et al. [16] enrolled 20 patients in a prospective, single-arm, multicenter, first-in-human study to evaluate the safety and performance of the Trillium stent graft system. Trillium is a cross-caval covered stent graft with a valved wall that spans the RA, with one end fixed in the SVC and the other in the IVC. The procedural success rate was 100%, with one case of mild paravalvular IVC leak. The most frequent 30-day complications were renal failure requiring hemodialysis (10%) and major gastrointestinal (GI) bleeding requiring intervention or blood transfusion (10%). At 30 days, TR was reduced in all patients (100%) from severe to mild. Conversely, RV function and dimensions were not affected by treatment, unlike left ventricle (LV) function and dimensions. Improvement in NYHA class was observed in 50% of patients (to NYHA class I or II), and a reduction in the edema severity score was observed in 75% of patients (to score I or II). Likewise, the KCCQ score increased after 30 days. Finally, the 30-day mortality was 5%; one patient died from progressive HF and multiorgan failure, while two patients (10%) were readmitted for HF.

Complete study details and all extracted data are available in **Supplementary Tables 4 and 5**.

## 4. Discussion

The development of right-sided HF and progressive TR is associated with increased mortality, more HF hospitalizations, and reduced quality of life. Most patients with severe TR have left-sided heart disease. Over time, tricuspid valve annular dilation and worsening TR cause progressive RV dilation and eventual failure, leading to increased RV diastolic pressure. In advanced, severe TR with RV failure, the interventricular septum shifts toward the LV, reducing LV cavity size and restricting LV filling. This further increases the already elevated LV diastolic and pulmonary artery (PA) pressures and likely exacerbates RV–PA uncoupling. The resulting elevation in LA and RA pressures can increase the risk of atrial fibrillation, irreversible RV failure, and death. Thus, earlier medical and procedural management of TR is essential to avoid deleterious downstream effects, such as cardiorenal and cardiohepatic syndromes, which can preclude candidacy for advanced therapies [17].

Current European guidelines [18] assign transcatheter tricuspid valve treatment to a class IIA recommendation for patients with symptomatic severe TR despite optimal medical therapy who are at high surgical risk, to improve quality of life and right ventricular remodeling, provided there is no severe RV dysfunction or precapillary pulmonary hypertension. Despite the availability of multiple transcatheter techniques, some patients are not suitable for T-TEER, annuloplasty, or orthotopic replacement. These may include patients with large annuli or tethered leaflets, multiple cardiac implantable electronic devices (CIEDs) crossing the tricuspid annulus, damaged leaflets, a large coaptation gap, pulmonary artery hypertension, and/or RV dysfunction. CAVI is feasible in patients with isolated TR and challenging anatomical features, such as pacemaker lead-induced TR, because the device does not interfere with the tricuspid valve.

Longstanding TR can lead to elevated central venous pressure, chronic volume overload, and many negative downstream effects. Concomitant renal and hepatic dysfunction are frequently encountered in patients with chronic severe TR. Elevated central venous pressure is transmitted to the renal veins, increasing renal venous pressure, decreasing renal blood flow, increasing interstitial pressure, and worsening renal function, thereby contributing to cardiorenal syndrome. Elevated central venous pressure is also transmitted to the hepatic veins, initiating a cascade of centrilobular congestion, sinusoidal dilation, and perivenular fibrosis. Centrilobular liver injury can extend peripherally with connective tissue deposition bridging the central veins and sometimes culminating in cardiac cirrhosis, also referred to as cardiohepatic syndrome. Finally, chronic volume overload can cause systemic congestion that overwhelms the splanchnic microcirculation, elevating intra-abdominal pressure. This may further reduce transmural perfusion pressures in the kidneys and liver, thereby worsening end-organ function [17]. The main purpose of CAVI is to prevent regurgitant flow from the right atrium into the IVC and SVC by placing one valve in each vena cava. This may reduce hepatic and renal congestion, thereby improving liver and kidney function and reducing ascites, abdominal congestion, and peripheral edema. In addition, decreasing tricuspid regurgitant flow increases RV forward stroke volume to the pulmonary circulation, because regurgitation diverts part of the total RV stroke volume away from effective pulmonary flow [19], thereby increasing oxygenated blood flow and improving cardiac output. In the long term, reducing right heart volume overload may promote reverse remodeling of the RV, reduce tricuspid annular dilatation, and improve TR severity. Reducing right heart volume overload may also improve RV functional parameters and address dysfunction. Overall, CAVI can improve the global functional status of patients, reducing NYHA class, dyspnea, diuretic doses, and hospitalization rates, while improving quality of life.

In this setting, CAVI was initially performed as compassionate use in patients who had no other transcatheter options and were at high surgical risk. The first preclinical explorations began in the early 2000s; however, the technique gained clinical relevance with the pioneering work by Lauten et al. [20,21] in 2011 and 2014, who reported the first successful transcatheter CAVI using a custom-made self-expanding prosthesis. Meanwhile, prostheses designed for deployment in the aortic position have been the most widely used, including the Edwards SAPIEN XT and SAPIEN 3 [22], both balloon-expandable, which have been used off-label under compassionate-use programs or in early feasibility trials [12]. However, one RCT reported a high risk of prosthesis migration or stent displacement with the Edwards SAPIEN XT [6], likely due to the contrast between the calcified aortic root in TAVR patients and the smooth luminal surface of the IVC in CAVI patients. Subsequent development accelerated with the introduction of the TricValve (P&F Products Features) and TRICENTO system (Medira AG, Balingen, Germany), two prostheses specifically created for the IVC and SVC. The TricValve is a transcatheter bicaval valve system consisting of two self-expanding biological valves implanted in the SVC and IVC without disturbing the native tricuspid valve. This procedure aims to decrease systemic venous congestion, particularly hepatic congestion, thereby reducing the extracardiac manifestations of severe TR and improving quality of life and functional status. The TRICENTO system is a transcatheter bicaval valved stent graft consisting of a self-expanding nitinol frame internally covered with thin porcine pericardium. The system is custom-made according to the anatomy of each patient and was first implemented in 2018 by Toggweiler et al. [23] and later by Montorfano et al. [24]. The device is intended to prevent backflow into the venous system, thereby reducing TR-related congestive symptoms. Owing to the individualized design of the system, this approach may overcome the anatomic and technical limitations of other transcatheter systems. Lurz et al. [16] also reported promising results with another stented valve, the Trillium device.

Wilbring et al. [25] described two cases of unsuccessful CAVI, with 100% procedural success but recurrent episodes of HF within 3 months after implantation. Four-dimensional magnetic resonance imaging (4D-MRI) showed nearly complete systolic compression of the stent graft in the RA, resulting in persistent systemic backflow and signs of venous congestion. In addition, persistent TR led to functional ventricularization of the RA. Hence, persistent systemic backflow and TR reduced RV autoregulation, leading to recurrent HF episodes due to the vulnerability of the right heart chambers to changes in volume load. RA ventricularization with persistently elevated NT-proBNP was also reported in other studies [8,12], although without a clear clinical correlation.

Currently, only one RCT of CAVI is available for the balloon-expandable Edwards XT valve; however, this study was interrupted due to a high rate of valve dislocation and conversion to open surgery. The lack of native calcified structures to anchor the device may lead to paravalvular leak or valve migration, which can potentially result in hemodynamic deterioration and even sudden cardiac death. Therefore, the only RCT of CAVI yielded unfavorable results.

**Fig. 2.**
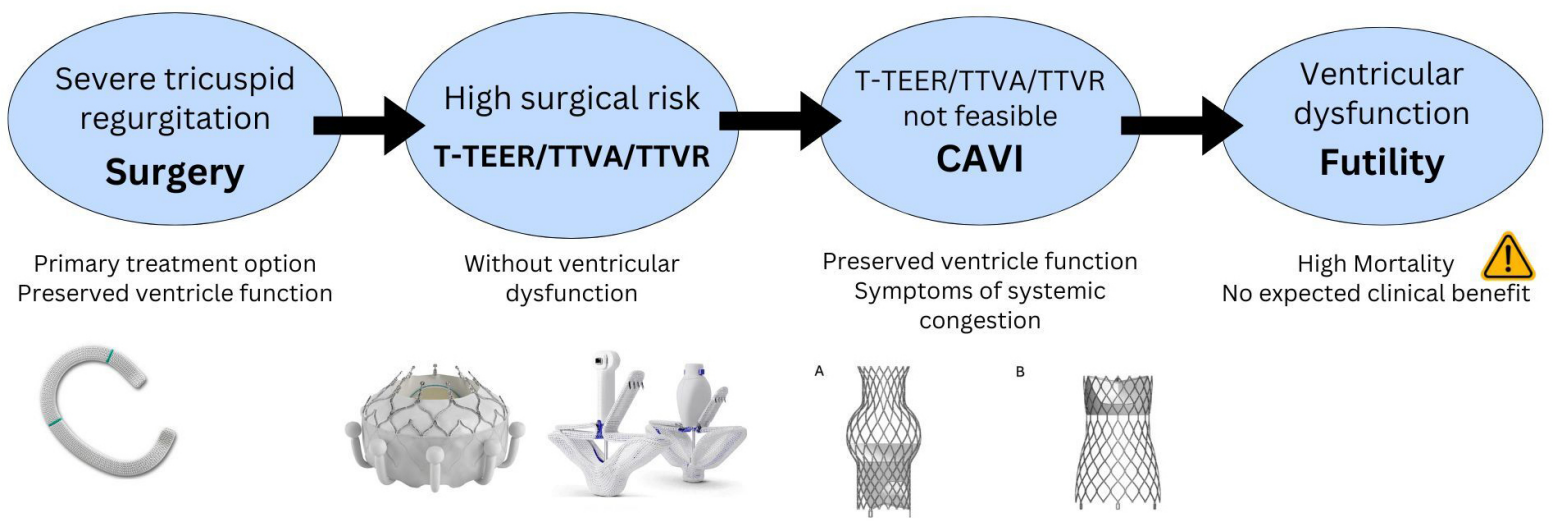
**Staged treatment options for severe tricuspid regurgitation**. CAVI, caval valve implantation; T-TEER, transcatheter tricuspid edge-to-edge repair; TTVA, transcatheter tricuspid valve annuloplasty; TTVR, transcatheter tricuspid valve replacement. (A) Superior Vena Cava Valve; (B) Inferior Vena Cava Valve. The image was created using the Canva graphic design platform.

Di Mauro et al. [14] demonstrated that end-stage patients with a high TRI-SCORE score may not benefit from this procedure, underscoring the importance of careful patient selection. According to the criteria proposed by Estévez-Loureiro et al. [8], CAVI should be considered in patients with severe or torrential TR who are not candidates for surgical or transcatheter direct tricuspid interventions. Patient selection relies mainly on clinical signs of systemic venous congestion and suitable caval anatomy rather than on tricuspid leaflet morphology or coaptation gap size. However, unfavorable anatomical features include caval thrombosis or severe stenosis, inadequate landing zones, excessive angulation between the vena cava and the RA, and a short distance from the hepatic veins, which may increase the risk of hepatic outflow obstruction. In addition, advanced RV failure, severe pulmonary hypertension, and end-stage hepatic dysfunction represent relative contraindications, as these issues are associated with limited clinical benefit and worse outcomes. Therefore, CAVI represents a heterotopic, palliative strategy aimed at reducing venous backflow and improving symptoms rather than correcting tricuspid valve incompetence (Fig. 2).

Sharkey et al. [26] described two patients with TR who were unsuitable for surgical intervention and underwent transcatheter heterotopic CAVI with the Edwards SAPIEN 3 valve. Both patients died within 12 months of the procedure, one from cardiogenic shock and sepsis, and the other from aspiration pneumonia, at 11 months and 9 months, respectively. Despite improvement in HF symptoms and renal function after the procedure, both ultimately died from other comorbid conditions. Similarly, Lauten et al. [12], in a multicenter registry study, reported a 1-year mortality of 63%, suggesting that heterotopic CAVI may function primarily as a palliative procedure to prevent and treat downstream complications and symptoms associated with severe, refractory TR.

O’Neill et al. [7] raised concerns about the futility of CAVI, noting that some patients are unlikely to derive measurable benefit. Patient risk factors associated with increased mortality after TAVR have been identified [27], and analogous risk factors will need to be identified and integrated for patients with severe TR to determine who may benefit from percutaneous therapies. In addition to survival and functional status, futility in transcatheter valve interventions also includes persistent HF symptoms, lack of reverse cardiac remodeling, and progression of end-organ dysfunction despite valve correction [28]. Accumulating evidence from TAVR has demonstrated that advanced frailty, severe comorbidity burden, and limited physiological reserve are key determinants of poor postprocedural outcomes, even when optimal device deployment is achieved. Therefore, despite promising hemodynamic improvements, CAVI may be futile in patients with advanced multiorgan dysfunction or severe, irreversible RV impairment. Evidence suggests that in the presence of profound hepatic congestion, renal failure, or elevated venous congestion scores, the procedural benefit may be limited because systemic congestion cannot be fully relieved, and end-organ recovery is unlikely. Our systematic review highlights that while CAVI shows promising hemodynamic and symptomatic benefits in selected patients, the risk of futility remains a critical consideration. As emphasized in studies of transcatheter tricuspid valve interventions (TTVIs), inappropriate timing or patient selection, particularly in the presence of advanced comorbidities, may result in minimal clinical improvement despite technically successful procedures [29]. These findings underscore the need to integrate comprehensive clinical, echocardiographic, and hemodynamic assessment into patient selection for CAVI. Future research should focus on developing validated predictive models to identify patients at high risk of futility, thereby optimizing procedural benefit and resource allocation.

In addition to the TRICUS study (NCT03723239), the TRICUS study Euro (NCT04141137), and TRICAVAL (NCT02387697), several studies in the CAVI field may influence future referral recommendations, including the Innoventric Trillium Stent Graft First-in-Human study (NCT04289870); Retrospective Prospective Multicentric Clinical Follow-up of Patients After Being Treated With TricValve® (NCT05114850); European Registry of Transcatheter Repair for Tricuspid Regurgitation (EuroTR, NCT06307262); TRICAV-II Pivotal: TRIcvalve biCAVal ventilsystem for severe TR (NCT06458907). Details of these studies are reported in **Supplementary Table 6**.

**Fig. 3.**
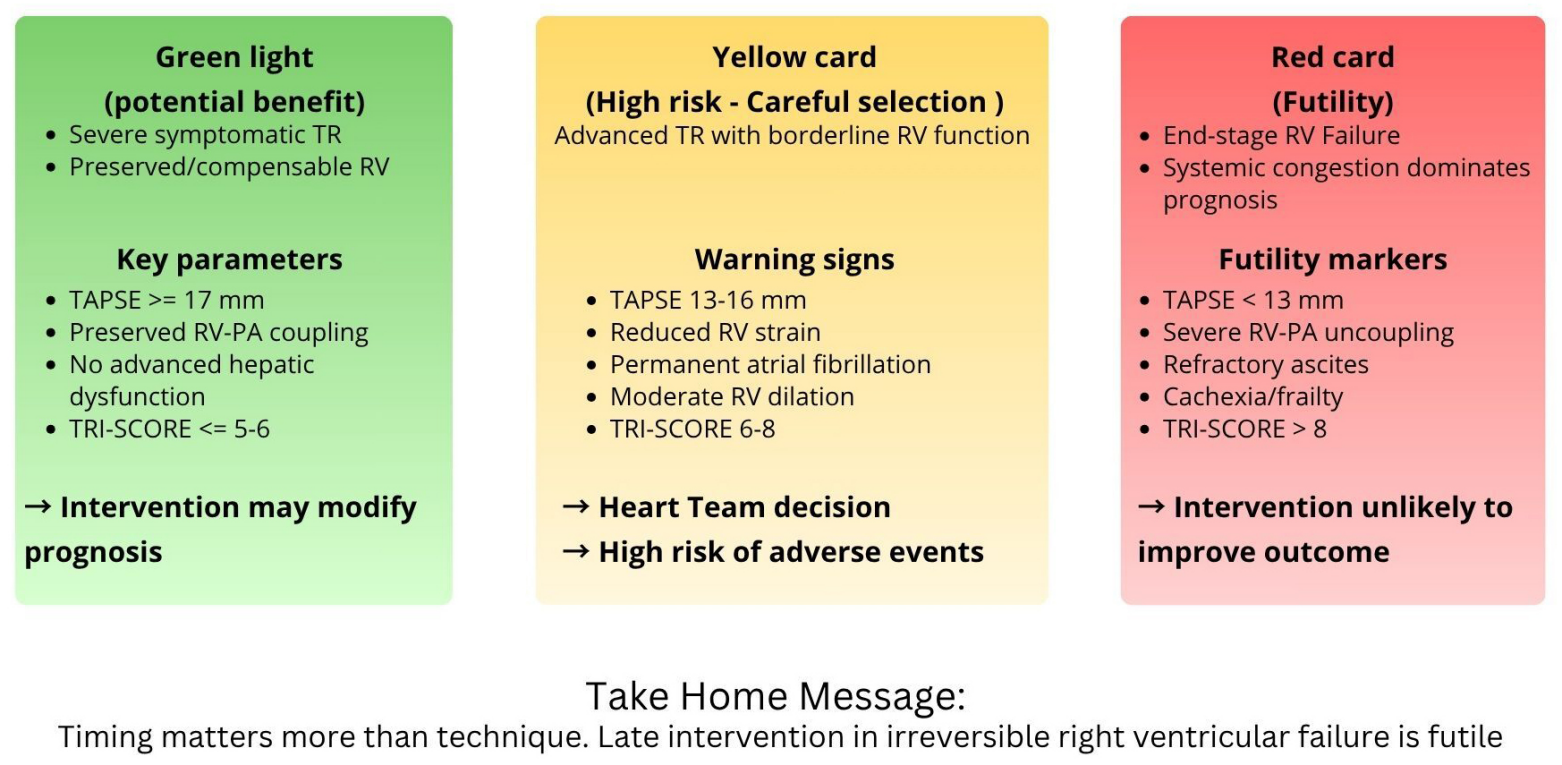
**Key take-home messages**. TR, tricuspid regurgitation; RV, right ventricle; TAPSE, tricuspid annular plane systolic excursion; PA, pulmonary artery. The image was created using the Canva graphic design platform.

Therefore, careful patient selection, incorporating objective metrics such as organ function markers, RV performance, and TRI-SCORE, is critical to avoid interventions unlikely to improve outcomes. In this context, futility should be regarded as a dynamic, multidimensional concept rather than a binary outcome, requiring the integration of clinical status, functional capacity, and patient-centered goals of care (Fig. 3).

## 5. Predictors and Determinants of Futility

### 5.1 Hemodynamic and Echocardiographic Factors

High pulmonary artery pressures and advanced RV dysfunction consistently predict poor outcomes. Estévez-Loureiro et al. [8], Blasco-Turrión et al. [10], and Sánchez-Recalde et al. [11], among registry studies, suggested avoiding CAVI when pulmonary artery systolic pressure (PASP) exceeds 65 mmHg, in the presence of pulmonary hypertension, or when severe RV dysfunction is present, defined as TAPSE <13 mm. Following TTVI, there is an expectation of reverse remodeling of the RV. However, the magnitude of RV reverse remodeling may be limited by pre-existing RV contractile dysfunction, the degree of residual or recurrent TR, pulmonary vascular resistance, and the severity of coexisting left-sided heart disease. LV filling, stroke volume (SV), and cardiac index may improve after TTVI for isolated TR due to favorable effects on ventricular interdependence and a rightward septal shift following a reduction in RV size [17].

PASP represents a key marker of RV afterload and plays a central role in the pathophysiology of advanced TR. Chronic elevation of PASP leads to progressive RV remodeling and dysfunction, ultimately resulting in RV–PA uncoupling, a condition in which the contractile reserve of the RV is no longer sufficient to accommodate the imposed afterload. RV–PA coupling, commonly assessed by the TAPSE/PASP ratio, has emerged as a robust, noninvasive surrogate of RV efficiency and a prognostic marker in HF and valvular heart disease [30]. RV–PA coupling refers to the ability of RV systolic performance to adapt to a given pulmonary afterload while maintaining adequate cardiac output. Furthermore, RV–PA coupling can be measured invasively or approximated using echocardiography by measuring TAPSE/PASP. A low TAPSE/PASP ratio occurs when afterload elevation can no longer be matched by RV contractile reserve and has been associated with a poor prognosis in different HF conditions, including severe TR [18]. This ratio has previously been linked to prognosis across HF phenotypes. Berthelot et al. [31] demonstrated that patients with HF with reduced ejection fraction (HFrEF) and a TAPSE/PASP ratio <0.40 mm/mmHg had significantly worse outcomes. In the 2022 European Society of Cardiology/European Respiratory Society guidelines [32], the TAPSE/PASP ratio is considered one of the determinants of prognosis in the risk assessment of pulmonary arterial hypertension (PAH) (Table 1). Tello et al. [33] demonstrated that the TAPSE/PASP ratio is associated with TR severity. This finding is of clinical value because severe TR *per se* has been identified as an independent prognostic feature in patients with PAH. However, TAPSE might be overestimated in the presence of severe TR, and severe TR has also been reported to influence the relationship between TAPSE and right ventricle ejection fraction (RVEF). The maladaptive morphological changes of RA/RV chamber enlargement that cause annular dilation and leaflet tethering, leading to TR in patients with PAH, are also associated with the TAPSE/PASP ratio. The TAPSE/PASP ratio has also been linked to interleukin-6 (IL-6), a relevant biomarker of RV function. Serum IL-6 levels are independently associated with RV function and RV–PA coupling in PAH. Patients with higher IL-6 levels exhibit more severe RV dysfunction and diminished RV–PA coupling despite comparable pulmonary vascular disease severity [34]. In severe TR, a TAPSE/PASP ratio <0.31 mm/mmHg is associated with poor prognosis [35,36]. Brener et al. [37] calculated RV–PA coupling ratios in patients enrolled in the global TriValve registry using TAPSE/PASP measurements from transthoracic echocardiography performed before and 30 days after the procedure. In that analysis, a low baseline TAPSE/PASP ratio (<0.406 mm/mmHg), which was associated with a higher prevalence of diabetes, previous myocardial infarction, and renal dysfunction, may be a marker of more chronic disease or later presentation with permanent maladaptation. Thus, in this subgroup, RV function may already be significantly reduced, remain unchanged following TTVR, and contribute to increased all-cause mortality. Furthermore, patients with a high baseline TAPSE/PASP ratio (>0.406 mm/mmHg) may have more afterload reserve to tolerate the abrupt increase in afterload following TTVR. In these patients, an increase in the RV–PA coupling ratio may indicate an appropriate ventricular and arterial response to TR reduction. Hence, RV–PA coupling is a potent hemodynamic biomarker associated with all-cause mortality in patients with TR who are undergoing TTVR. These findings may help evaluate the benefit of TTVR by setting a TAPSE/PASP cutoff of 0.31 mm/mmHg, which discriminates RV–PA uncoupling with a sensitivity of 87.5% and a specificity of 75.9%, and is independently associated with prognosis [37]. Gavazzoni et al. [38] demonstrated that both TR severity and echocardiographic RV–PA coupling indices are strongly and independently associated with the occurrence of the composite end point of all-cause death and hospitalization for HF. This study described a new three-dimensional (3D) echocardiographic index obtained by adapting the formula of invasive coupling to account for the regurgitant volume of TR (*i*.*e*., RV forward SV/end-systolic volume (ESV)), which showed a closer association with outcome than other echocardiographic RV–PA coupling indices, such as TAPSE/PASP, RVEF, and right ventricle free wall longitudinal strain (RVFWLS)/PASP. However, the TAPSE/PASP threshold was extrapolated from the HF and orthotopic tricuspid intervention literature. Thus, the threshold should be interpreted with caution and not considered a validated cutoff for predicting CAVI futility. Consequently, studies with larger patient cohorts undergoing CAVI and longer follow-up are needed to confirm these results and identify a TAPSE/PASP ratio cutoff applicable to CAVI.

**Table 1.** **Risk assessment of pulmonary arterial hypertension in the 2022 ESC/ERS guidelines**.

Determinants of prognosis (estimated 1-year mortality)	Low risk (<5%)	Intermediate risk (5–20%)	High risk (>20%)
TAPSE/PASP ratio	>0.32 mm/mmHg	0.32–0.19 mm/mmHg	<0.19 mm/mmHg

TAPSE/PASP, tricuspid annular plane systolic excursion/pulmonary artery systolic pressure; ESC/ERS, European Society of Cardiology/European Respiratory Society.

In the context of heterotopic CAVI, elevated PASP and impaired RV–PA coupling have major implications for procedural futility. Although CAVI effectively reduces systemic venous congestion by preventing caval backflow and reducing RV pressure, this approach does not directly address the underlying RV dysfunction or pulmonary vascular disease. In patients with normal RV function, CAVI reduces RV preload, lowering RV pressure and, consequently, RV stroke work and oxygen consumption, resulting in improved RV function. In patients with advanced RV failure and RV–PA uncoupling, the abrupt hemodynamic changes following bicaval valve implantation, namely increased effective RV preload and loss of the “pop-off” mechanism provided by severe TR, may overwhelm a failing RV, precipitating low-output states or persistent right-sided HF. Several observational studies and early registries have shown that patients with severely impaired RV function, high PASP, and evidence of RV–PA uncoupling derive limited clinical benefit from CAVI, with persistent symptoms and poor mid-term outcomes despite technical procedural success [12,39]. Consequently, the integration of PASP and RV–PA coupling metrics into preprocedural evaluation is increasingly recognized as essential for identifying patients in whom CAVI may represent a futile intervention rather than a meaningful therapeutic strategy.

Severe LV dysfunction (LVEF <35%) and fixed RV dilation further diminish the hemodynamic benefit. Recent evidence from the international TRIGISTRY registry [40] highlights the critical influence of baseline cardiac function on the potential benefit of structural interventions in right-sided heart disease. In patients with severe TR, transcatheter or surgical valve intervention conferred a clear survival advantage over conservative management only in those with preserved LVEF, whereas this benefit was absent in those with mildly or severely reduced LVEF. Moreover, residual TR after intervention was consistently associated with worse outcomes across the entire LVEF spectrum, further underscoring that procedural success is a key determinant of clinical benefit. These findings elegantly illustrate the futility of structural interventions in the setting of advanced ventricular dysfunction, where correcting the valvular lesion alone fails to translate into improved survival, and where the underlying myocardial substrate and systemic congestion, including end-organ impairment, may be the dominant drivers of poor prognosis. Such data reinforce the need for robust preprocedural stratification, integrating ventricular performance, congestion burden, and end-organ metrics to identify patients most likely to derive meaningful benefit from CAVI and similar transcatheter therapies.

### 5.2 End-Organ Damage

Hepatic and renal dysfunction are strong independent predictors of mortality. Patients with elevated bilirubin or creatinine often exhibit limited symptomatic improvement even when caval reflux is reduced, reflecting irreversible systemic venous and microcirculatory damage or permanent organ damage due to prolonged venous congestion. Estévez-Loureiro et al. [8] and Dreger et al. [6] suggested avoiding CAVI in patients with significant renal dysfunction (creatinine >3 mg/dL), dialysis within the previous 4 weeks, or dialysis at the time of screening.

The venous excess ultrasound (VExUS) score [41] is a composite sonographic tool designed to quantify systemic venous congestion by integrating IVC diameter with Doppler flow patterns in the hepatic, portal, and intrarenal veins. Originally developed to predict acute kidney injury in patients undergoing cardiac surgery, VExUS grading has since been explored across a range of cardiovascular conditions, including acute and chronic HF, where congestion severity has strong prognostic implications [42,43]. In structural heart disease, systemic venous congestion is a key mediator of end-organ dysfunction and an independent determinant of outcomes. A high VExUS score reflects significant transmission of elevated RA pressures into the hepatic and renal venous circulations, which correlates with renal impairment and adverse short-term outcomes in HF cohorts [44]. Importantly, VExUS has demonstrated superior specificity for predicting elevated filling pressures than IVC diameter alone and shows a strong association with invasively measured RA pressure, supporting the physiological relevance of the metric in right-sided hemodynamic assessment [45]. In the CAVI population, VExUS could enhance preprocedural stratification by providing a noninvasive marker of systemic congestion burden beyond traditional echocardiographic or clinical metrics. Recent work has shown that subclinical venous congestion assessed by VExUS at hospital discharge, despite apparent euvolemia, is prevalent and independently predicts adverse outcomes at 6 months, highlighting the prognostic relevance of residual congestion that may be missed by conventional clinical assessment [46]. Given that persistent venous congestion is implicated in futility scenarios, where relieving regurgitation alone does not translate into meaningful organ recovery, integrating VExUS into clinical workflows may help identify patients with advanced congestion phenotypes unlikely to benefit from CAVI. Such integration aligns with emerging evidence that sonographic congestion assessment outperforms single parameters in risk stratification and could refine selection criteria in patients with coexisting hepatic or renal dysfunction [47]. VExUS could also be used to quantify reductions in systemic congestion before and after the procedure, providing an objective measure of congestion improvement. Unfortunately, VExUS is neither reported nor considered in the studies included in this systematic review nor in the literature. Therefore, VExUS remains a promising but unvalidated assessment tool. Further studies are needed to apply this metric in the field of CAVI and validate this score for measuring congestion improvement in patients undergoing CAVI.

### 5.3 Clinical Frailty and Comorbidities

Advanced age, frailty, cachexia, and low albumin are associated with high postprocedural mortality. In such cases, the intervention may relieve congestion temporarily but fail to alter the disease trajectory and may lead to death. Moreover, the prevalence of TR increases with age; thus, patients considered for CAVI are generally older than patients with other cardiac pathologies, and are often considered unsuitable for surgery due to high surgical risk related to comorbidities and other conditions associated with aging. Frailty and the burden of comorbidities are increasingly recognized as critical determinants of procedural futility in structural heart interventions. In patients considered for CAVI, frailty, characterized by impaired mobility, sarcopenia, and diminished physiological reserve, has been consistently associated with worse postprocedural outcomes, prolonged recovery, and reduced survival benefit, independent of valvular hemodynamic severity. Similarly, coexisting conditions such as advanced chronic kidney disease, significant hepatic dysfunction, severe pulmonary hypertension, or chronic lung disease may limit the capacity of patients to tolerate the hemodynamic shifts induced by CAVI and blunt the expected improvement in systemic venous congestion. Collectively, frailty and a high comorbidity burden define a phenotype in which intervention may be technically successful yet clinically futile, as the underlying vulnerability precludes meaningful functional recovery. Common instruments for evaluating frailty include the Clinical Frailty Scale (CFS) [48], a 9-point judgement-based scale summarizing functional status and comorbidity burden; the Fried frailty phenotype [49], which operationalizes frailty as a physical syndrome defined by weight loss, weakness, slowness, exhaustion and low activity; the frailty index (FI) [50], which quantifies the proportion of accumulated health deficits. Validated across diverse clinical contexts, these scores predict mortality and adverse outcomes beyond those predicted by traditional comorbidity measures. For quantifying comorbidity burden specifically, indices such as the Charlson comorbidity index (CCI) [51] and the Elixhauser comorbidity index [52] are widely used to estimate long-term mortality risk and to contextualize frailty in the setting of multimorbidity. Integrating validated frailty scores and comprehensive comorbidity indices into preprocedural assessment allows for more nuanced risk stratification, complementing hemodynamic and congestion metrics such as the VExUS score, and helps identify patients unlikely to derive substantial benefit from CAVI. Therefore, CAVI may be avoided in the presence of any life-threatening non-cardiac disease or a life expectancy of <1 year, as proposed by Blasco-Turrión et al. [10] and Dreger et al. [6].

### 5.4 Anatomical and Procedural Considerations

Device-specific anatomical considerations should be carefully evaluated when assessing candidates for CAVI, as currently available systems differ substantially in design, landing zones, and interaction with venous inflow. Dedicated bicaval systems such as TricValve are specifically engineered for deployment in both the SVC and IVC and require adequate caval diameters, sufficient landing zone length, and careful assessment of the relationship with hepatic vein inflow to avoid obstruction. In contrast, custom-made devices such as TRICENTO are tailored to patient-specific anatomy, allowing treatment of complex geometries but requiring precise preprocedural imaging and planning. Balloon-expandable valves (*e*.*g*., Edwards SAPIEN) have been used off-label in the vena cava, often necessitating pre-stenting to create a stable anchoring zone; thus, these valves are limited by caval size and compliance, as well as by the risk of migration or paravalvular leak. Balloon-expandable valves generally require diameters between 18 and 28 mm. Extreme caval dilation (>30–32 mm) may preclude safe anchoring and is usually considered unfavorable for this approach. Incomplete sealing of the caval prostheses, underexpansion, or device migration can result in residual reflux, negating symptomatic benefit. For TricValve, the mean IVC diameter must be measured at end-diastole, and a diameter >30–34 mm is an exclusion criterion due to insufficient anchoring and migration risk. A marked ellipticity of the IVC is associated with a higher risk of paravalvular leak or instability. In addition, excessive collapse or expansion between inspiration and expiration is considered unfavorable. The distance from the RA junction to the hepatic vein confluence must be >30–40 mm (device-dependent). A shorter distance is associated with a higher risk of hepatic vein obstruction. The ostia of the hepatic veins must be >10–15 mm below the inferior edge of the prosthesis. Exclusion criteria include high takeoff hepatic veins, multiple hepatic veins with a short common trunk, and direct hepatic vein drainage into the right atrium. In bicaval CAVI, the typical acceptable diameter range for the SVC is 16–28 mm, while the minimum SVC landing zone must be ≥25–30 mm, with an adequate distance from the brachiocephalic vein confluence and the azygos vein ostium. In cases of excessive RA dilation, there is a high risk of device instability, whereas in very small RA cases, there is a high risk of interference with atrial structures. Therefore, only patients with severe TR or higher grades (massive or torrential) should be considered for CAVI. Furthermore, assessing device protrusion into the RA is important for evaluating the risk of compression of the sinoatrial node region, hepatic veins, and adjacent vascular structures. Finally, the femoral vein diameter must accommodate 22–27 Fr sheaths (device-specific). Exclusion criteria for unsuitable femoral access include severe venous tortuosity, caval thrombosis, and heavy calcification. Careful computed tomography (CT)-based planning with contrast-enhanced ECG-gated multidetector computed tomography (MDCT) is essential to minimize technical futility.

### 5.5 Risk Scores

In the TAVR setting, futility has traditionally been associated with early mortality or lack of improvement in functional status and quality of life, underscoring that valve correction alone may be insufficient in patients with advanced myocardial disease, frailty, or end-organ dysfunction [27]. In transcatheter valve procedures, futility refers to the absence of meaningful clinical benefit, in terms of survival, symptom improvement, or quality of life, despite a technically successful intervention. Although no universally accepted definition exists, many analyses in the TAVI field define futility using 1-year mortality or a composite endpoint of mortality and lack of functional improvement as reference outcomes for ineffective interventions [53]. Similar concerns are increasingly emerging in TTVI. In this field, the issue of futility is particularly relevant given the pattern of late referrals and the frequent coexistence of advanced right-sided HF. Recent evidence in TTVI has highlighted the limitations of conventional surgical risk scores and the added value of disease-specific tools such as the TRI-SCORE [54], which integrates clinical, laboratory, and echocardiographic markers of right-sided HF and end-organ dysfunction. In real-world cohorts, a high TRI-SCORE (≥8) has been consistently associated with increased mortality and hospitalization for HF, irrespective of procedural success, suggesting that anatomical correction alone may be insufficient once advanced myocardial and systemic impairment is established. Notably, high TRI-SCORE values have been associated with increased mortality and HF hospitalization irrespective of procedural success, suggesting that correction of TR may come too late in the disease course to promote a meaningful alteration to prognosis [55]. Importantly, these findings support the notion that futility should not be defined solely by technical feasibility or acute hemodynamic improvement, but rather by the global stage of disease at the time of intervention. This concept is well established in the surgical management of isolated TR, where perioperative counseling, identification of patients suitable for tricuspid valve surgery, and early referral for surgical intervention are crucial to avoid unnecessary surgical risk [56]. Translating this paradigm to complex transcatheter valve therapies, including CAVI, underscores the need for early referral and comprehensive risk stratification to identify a therapeutic “sweet spot” where intervention can modify the prognosis rather than merely palliate symptoms [57]. This paradigm emphasizes the need for early referral, comprehensive multimodality assessment, and careful patient selection to avoid interventions that offer limited prognostic or symptomatic benefit. Such considerations are highly relevant when extending transcatheter therapies to advanced clinical scenarios, including CAVI, where systemic venous congestion and multisystem involvement may further amplify the risk of futility.

## 6. Limits and Future Perspective

Defining objective criteria for futility will require integration of:

• Quantitative imaging: integration of RV strain, 3D volumes, and coupling indices with RV and LV function, as well as identification of cutoff values for selecting patients who may benefit from CAVI.

• Biomarkers: establishment of the prognostic and outcome-related role of NT-proBNP, bilirubin, albumin, and creatinine, to identify patients with organ impairment and end-stage disease.

• Risk stratification tools: integration of TRI-SCORE, frailty indices, and multiorgan function assessment to avoid CAVI in patients at high risk of mortality despite the procedure.

This systematic review has several limitations that should be acknowledged. First, relevant studies may have been inadvertently omitted, and the relative weight of the evidence may not be uniformly represented. Second, the available evidence on tricuspid valve disease, particularly in the CAVI field, is largely derived from early-phase trials, single-arm studies, registries, and observational cohorts, often with limited sample sizes and short-term follow-up. Therefore, the apparent benefits reported in subsequent registries must be interpreted with caution, given the observational design. Consequently, long-term durability, clinical outcomes, and device-related complications remain incompletely defined. Third, there is substantial heterogeneity in patient populations, definitions of TR severity, imaging criteria, and procedural endpoints across studies. This heterogeneity limits direct comparisons among studies and the interpretation of overall outcomes. Fourth, most published data focus on high-risk or inoperable patients, which restricts the generalizability of current findings to lower-risk populations or earlier stages of tricuspid valve disease. In addition, many studies lack standardized reporting of right ventricular function, RA remodeling, and end-organ involvement, which are critical determinants of outcome. Fifth, rapid technological evolution and continuous refinement of devices and implantation techniques mean that some of the evidence discussed may quickly become outdated, and conclusions drawn from earlier-generation devices may not fully apply to current or future technologies. Finally, publication bias and expert opinion may influence the interpretation of the results, particularly in the CAVI field, where randomized controlled trials are lacking. Therefore, current conclusions are based mainly on observational evidence and expert-driven clinical interpretation, and should be considered hypothesis-generating rather than definitive. Ongoing registries, randomized clinical trials, and upcoming prospective studies will clarify whether early referral, before irreversible RV and end-organ deterioration, can expand the effective window for CAVI.

## 7. Conclusions

CAVI provides symptomatic and hemodynamic improvement in selected high-risk patients with severe TR, but the benefit of this approach is strongly dependent on baseline RV and systemic condition. When advanced pulmonary hypertension, irreversible RV failure, or multiorgan dysfunction are present, the intervention often becomes futile, offering little survival advantage despite technical success. A standardized, multidisciplinary evaluation combining hemodynamic, imaging, and clinical parameters is essential to distinguish patients likely to benefit from those in whom CAVI should be considered purely palliative. This procedure is often the last option for patients who are at high surgical risk and are unsuitable for other transcatheter techniques. In advanced TR, CAVI may shift from being a therapeutic to a palliative intervention, aimed primarily at improving symptoms rather than survival. Recognizing this transition requires open discussions about goals of care and shared decision-making. Procedural success should not overshadow the broader question of whether the intervention achieves outcomes that are meaningful to the values and expected lifespan of the patient. The definition of universal criteria for futility and the early assessment of severe tricuspid valve regurgitation, in the absence of severe RV and/or LV dysfunction and pulmonary hypertension without signs of irreversible organ damage, will lead to a selection of patients who will benefit from CAVI and improve survival in conjunction with quality of life, while excluding those who are too ill to benefit and are likely to die despite the intervention. Highlighting the futility does not undermine the potential of the technique but rather underscores the importance of individualized, evidence-based decision-making in high-risk populations. The art of CAVI lies as much in knowing when not to intervene as in performing the procedure well.

## Data Availability

This systematic review used data from previously published studies, all of which are available in the cited literature. No additional datasets were generated or analyzed.

## Abbreviations

CAVI, caval valve implantation; EF, ejection fraction; FAC, fractional area change; HF, heart failure; IVC, inferior vena cava; KCCQ, Kansas City Cardiomyopathy Questionnaire; LV, left ventricle; LVEF, left ventricular ejection fraction; MDCT, multidetector computed tomography; MRI, magnetic resonance imaging; NYHA, New York Heart Association; OMT, optimized medical therapy; PA, pulmonary artery; PASP, pulmonary artery systolic pressure; PMK, pacemaker; RA, right atrium; RV, right ventricle; SVC, superior vena cava; TAPSE, tricuspid annular plane systolic excursion; TAVR, transcatheter aortic valve replacement; TR, tricuspid regurgitation; T-TEER, transcatheter tricuspid edge-to-edge repair; TTVI, transcatheter tricuspid valve intervention; 6MWT, 6-minute walking test.

## References

[1] Topilsky Y, Maltais S, Medina Inojosa J, Oguz D, Michelena H, Maalouf J (2019). Burden of Tricuspid Regurgitation in Patients Diagnosed in the Community Setting. JACC. Cardiovascular Imaging.

[2] Dreyfus J, Flagiello M, Bazire B, Eggenspieler F, Viau F, Riant E (2020). Isolated tricuspid valve surgery: impact of aetiology and clinical presentation on outcomes. European Heart Journal.

[3] Zack CJ, Fender EA, Chandrashekar P, Reddy YNV, Bennett CE, Stulak JM (2017). National Trends and Outcomes in Isolated Tricuspid Valve Surgery. Journal of the American College of Cardiology.

[4] Di Mauro M, Bonalumi G, Giambuzzi I, Masiero G, Tarantini G (2025). Isolated tricuspid regurgitation: a new entity to face. Prevalence, prognosis and treatment of isolated tricuspid regurgitation. Minerva Cardiology and Angiology.

[5] Abdul-Jawad Altisent O, Benetis R, Rumbinaite E, Mizarien V, Codina P, Gual-Capllonch F (2021). Caval Valve Implantation (CAVI): An Emerging Therapy for Treating Severe Tricuspid Regurgitation. Journal of Clinical Medicine.

[6] Dreger H, Mattig I, Hewing B, Knebel F, Lauten A, Lembcke A (2020). Treatment of Severe TRIcuspid Regurgitation in Patients with Advanced Heart Failure with CAval Vein Implantation of the Edwards Sapien XT VALve (TRICAVAL): a randomised controlled trial. EuroIntervention : Journal of EuroPCR in Collaboration with the Working Group on Interventional Cardiology of the European Society of Cardiology.

[7] O'Neill BP, Negrotto S, Yu D, Lakhter V, Depta J, McCabe JM (2020). Caval Valve Implantation for Tricuspid Regurgitation: Insights From the United States Caval Valve Registry. The Journal of Invasive Cardiology.

[8] Estévez-Loureiro R, Sánchez-Recalde A, Amat-Santos IJ, Cruz-González I, Baz JA, Pascual I (2022). 6-Month Outcomes of the TricValve System in Patients With Tricuspid Regurgitation: The TRICUS EURO Study. JACC. Cardiovascular Interventions.

[9] Wild MG, Lubos E, Cruz-Gonzalez I, Amat-Santos I, Ancona M, Andreas M (2022). Early Clinical Experience With the TRICENTO Bicaval Valved Stent for Treatment of Symptomatic Severe Tricuspid Regurgitation: A Multicenter Registry. Circulation. Cardiovascular Interventions.

[10] Blasco-Turrión S, Briedis K, Estévez-Loureiro R, Sánchez-Recalde A, Cruz-González I, Pascual I (2024). Bicaval TricValve Implantation in Patients With Severe Symptomatic Tricuspid Regurgitation: 1-Year Follow-Up Outcomes. JACC. Cardiovascular Interventions.

[11] Sánchez-Recalde A, Domínguez-Rodríguez LM, Rosseel L, Nombela-Franco L, Pfister R, Amat-Santos I (2025). Bicaval TricValve Implantation in Patients With Severe Tricuspid Regurgitation: 1-Year Outcomes From the TricBicaval Registry. JACC. Cardiovascular Interventions.

[12] Lauten A, Figulla HR, Unbehaun A, Fam N, Schofer J, Doenst T (2018). Interventional Treatment of Severe Tricuspid Regurgitation: Early Clinical Experience in a Multicenter, Observational, First-in-Man Study. Circulation. Cardiovascular Interventions.

[13] Aalaei-Andabili SH, Bavry AA, Choi C, Arnaoutakis G, Anderson RD, Beaver TM (2020). Percutaneous Inferior Vena Cava Valve Implantation May Improve Tricuspid Valve Regurgitation and Cardiac Output: Lessons Learned. Innovations (Philadelphia, Pa.).

[14] Di Mauro M, Guarracini S, Mazzocchetti L, Capuzzi D, Salute L, Di Marco M (2024). Transcatheter bicaval valve system for the treatment of severe isolated tricuspid regurgitation. Features from a single-Centre experience. International Journal of Cardiology.

[15] O'Neill BP, Amoroso NS, Yadav P, Houston BA, Villablanca P, O'Neill WW (2025). Early Feasibility Study of the Edwards SAPIEN 3 Transcatheter Heart Valve System With the Edwards Caval Prestent for the Treatment of Reverse Caval Flow in Patients With Severe Tricuspid Regurgitation (TR). Catheterization and Cardiovascular Interventions : Official Journal of the Society for Cardiac Angiography & Interventions.

[16] Lurz P, Kresoja KP, Besler C, Verheye S, Vermeersch P, Rudolph V (2025). Heterotopic Crosscaval Transcatheter Tricuspid Valve Replacement for Patients With Tricuspid Regurgitation: The Trillium Device. JACC. Cardiovascular Interventions.

[17] O'Gara PT, Lindenfeld J, Hahn RT, Joseph M, Khalique OK, Khazanie P (2026). 10 Issues for the Clinician in Tricuspid Regurgitation Evaluation and Management: 2025 ACC Expert Consensus Decision Pathway: A Report of the American College of Cardiology Solution Set Oversight Committee. Journal of the American College of Cardiology.

[18] Praz F, Borger MA, Lanz J, Marin-Cuartas M, Abreu A, Adamo M (2025). 2025 ESC/EACTS Guidelines for the management of valvular heart disease: Developed by the task force for the management of valvular heart disease of the European Society of Cardiology (ESC) and the European Association for Cardio-Thoracic Surgery (EACTS). European Heart Journal.

[19] Sala A, Hahn RT, Kodali SK, Mack MJ, Maisano F (2023). Tricuspid Valve Regurgitation: Current Understanding and Novel Treatment Options. Journal of the Society for Cardiovascular Angiography & Interventions.

[20] Lauten A, Ferrari M, Hekmat K, Pfeifer R, Dannberg G, Ragoschke-Schumm A (2011). Heterotopic transcatheter tricuspid valve implantation: first-in-man application of a novel approach to tricuspid regurgitation. European Heart Journal.

[21] Lauten A, Doenst T, Hamadanchi A, Franz M, Figulla HR (2014). Percutaneous bicaval valve implantation for transcatheter treatment of tricuspid regurgitation: clinical observations and 12-month follow-up. Circulation. Cardiovascular Interventions.

[22] Laule M, Stangl V, Baumann G, Stangl K (2013). Reply: management of tricuspid regurgitation by caval valve implantation: from technical feasibility to evaluation of efficacy. Journal of the American College of Cardiology.

[23] Toggweiler S, De Boeck B, Brinkert M, Buhmann R, Bossard M, Kobza R (2018). First-in-man implantation of the Tricento transcatheter heart valve for the treatment of severe tricuspid regurgitation. EuroIntervention : Journal of EuroPCR in Collaboration with the Working Group on Interventional Cardiology of the European Society of Cardiology.

[24] Montorfano M, Beneduce A, Ancona MB, Ancona F, Sgura F, Romano V (2019). Tricento Transcatheter Heart Valve for Severe Tricuspid Regurgitation: Procedural Planning and Technical Aspects. JACC. Cardiovascular Interventions.

[25] Wilbring M, Tomala J, Ulbrich S, Murugaboopathy V, Matschke K, Kappert U (2020). Recurrence of Right Heart Failure After Heterotopic Tricuspid Intervention: A Conceptual Misunderstanding?. JACC. Cardiovascular Interventions.

[26] Sharkey A, Munoz Acuna R, Belani K, Sharma RK, Chaudhary O, Fatima H (2020). Heterotopic caval valve implantation for the management of severe tricuspid regurgitation: a case series. European Heart Journal. Case Reports.

[27] Lindman BR, Alexander KP, O'Gara PT, Afilalo J (2014). Futility, benefit, and transcatheter aortic valve replacement. JACC. Cardiovascular Interventions.

[28] Afilalo J, Lauck S, Kim DH, Lefèvre T, Piazza N, Lachapelle K (2017). Frailty in Older Adults Undergoing Aortic Valve Replacement: The FRAILTY-AVR Study. Journal of the American College of Cardiology.

[29] von Stein J, von Stein P, Alu MC, Scotti A, Ho EC, Granada JF (2025). Patient Selection for Transcatheter Tricuspid Valve Intervention: Not Too Early, Not Too Late. Structural Heart : the Journal of the Heart Team.

[30] Guazzi M, Bandera F, Pelissero G, Castelvecchio S, Menicanti L, Ghio S (2013). Tricuspid annular plane systolic excursion and pulmonary arterial systolic pressure relationship in heart failure: an index of right ventricular contractile function and prognosis. American Journal of Physiology. Heart and Circulatory Physiology.

[31] Berthelot E, Bauer F, Fauvel C, Paclot M, Eicher JC, de Groote P (2026). Echocardiographic risk stratification in heart failure with post-capillary pulmonary hypertension: prognostic value of LAVI and TAPSE/PASP. European Heart Journal. Cardiovascular Imaging.

[32] Humbert M, Kovacs G, Hoeper MM, Badagliacca R, Berger RM, Brida M (2022). 2022 ESC/ERS Guidelines for the diagnosis and treatment of pulmonary hypertension: Developed by the task force for the diagnosis and treatment of pulmonary hypertension of the European Society of Cardiology (ESC) and the European Respiratory Society (ERS). Endorsed by the International Society for Heart and Lung Transplantation (ISHLT) and the European Reference Network on rare respiratory diseases (ERN-LUNG). European Heart Journal.

[33] Tello K, Axmann J, Ghofrani HA, Naeije R, Narcin N, Rieth A (2018). Relevance of the TAPSE/PASP ratio in pulmonary arterial hypertension. International Journal of Cardiology.

[34] Prins KW, Archer SL, Pritzker M, Rose L, Weir EK, Sharma A (2018). Interleukin-6 is independently associated with right ventricular function in pulmonary arterial hypertension. The Journal of Heart and Lung Transplantation : the Official Publication of the International Society for Heart Transplantation.

[35] Barbato R, Loreni F, Ferrisi C, Mastroianni C, D'Ascoli R, Nenna A (2025). Isolated Tricuspid Regurgitation: When Is Surgery Appropriate? A State-of-the-Art Narrative Review. Journal of Clinical Medicine.

[36] Tello K, Wan J, Dalmer A, Vanderpool R, Ghofrani HA, Naeije R (2019). Validation of the Tricuspid Annular Plane Systolic Excursion/Systolic Pulmonary Artery Pressure Ratio for the Assessment of Right Ventricular-Arterial Coupling in Severe Pulmonary Hypertension. Circulation. Cardiovascular Imaging.

[37] Brener MI, Lurz P, Hausleiter J, Rodés-Cabau J, Fam N, Kodali SK (2022). Right Ventricular-Pulmonary Arterial Coupling and Afterload Reserve in Patients Undergoing Transcatheter Tricuspid Valve Repair. Journal of the American College of Cardiology.

[38] Gavazzoni M, Badano LP, Cascella A, Heilbron F, Tomaselli M, Caravita S (2023). Clinical Value of a Novel Three-Dimensional Echocardiography-Derived Index of Right Ventricle-Pulmonary Artery Coupling in Tricuspid Regurgitation. Journal of the American Society of Echocardiography : Official Publication of the American Society of Echocardiography.

[39] Kodali S, Hahn RT, Eleid MF, Kipperman R, Smith R, Lim DS (2021). Feasibility Study of the Transcatheter Valve Repair System for Severe Tricuspid Regurgitation. Journal of the American College of Cardiology.

[40] Heitzinger G, Dreyfus J, Dannenberg V, Topilsky Y, Benfari G, Marsan NA (2025). Left ventricular ejection fraction and benefit of tricuspid valve interventions - insights from the international TRIGISTRY. European Journal of Heart Failure.

[41] Beaubien-Souligny W, Rola P, Haycock K, Bouchard J, Lamarche Y, Spiegel R (2020). Quantifying systemic congestion with Point-Of-Care ultrasound: development of the venous excess ultrasound grading system. The Ultrasound Journal.

[42] Soliman D, Puchongmart C, Thiravetyan B, Cruz D, Yanpiset P, Ortiz Maldonado A (2026). Applications of Venous Excess Ultrasound Score (VExUS) in Volume Status Assessment in Patients With Acute Decompensated Heart Failure and Cardiorenal Syndrome. Cardiology in Review.

[43] Borroni VN, Antonini A, Cogliati CB, Casella F (2026). A comprehensive review of venous excess ultrasound (VExUS) score: evidence from original research. Internal and Emergency Medicine.

[44] Landi I, Guerritore L, Iannaccone A, Ricotti A, Rola P, Garrone M (2024). Assessment of venous congestion with venous excess ultrasound score in the prognosis of acute heart failure in the emergency department: a prospective study. European Heart Journal Open.

[45] Longino A, Martin K, Leyba K, Siegel G, Gill E, Douglas IS (2023). Correlation between the VExUS score and right atrial pressure: a pilot prospective observational study. Critical Care (London, England).

[46] Lozano-Jiménez S, García Sebastian C, Vela Martín P, García Magallón B, Martín Centellas A, de Castro D (2026). Prevalence and prognostic impact of subclinical venous congestion in patients hospitalized for acute heart failure. European Heart Journal. Acute Cardiovascular Care.

[47] Pierre-Grégoire G (2025). VExUS score: optimizing its use in perioperative and critical care management. Critical Care (London, England).

[48] Deng Y, Sato N (2024). Global frailty screening tools: Review and application of frailty screening tools from 2001 to 2023. Intractable & Rare Diseases Research.

[49] Le Pogam MA, Seematter-Bagnoud L, Niemi T, Assouline D, Gross N, Trächsel B (2022). Development and validation of a knowledge-based score to predict Fried's frailty phenotype across multiple settings using one-year hospital discharge data: The electronic frailty score. EClinicalMedicine.

[50] Searle SD, Mitnitski A, Gahbauer EA, Gill TM, Rockwood K (2008). A standard procedure for creating a frailty index. BMC Geriatrics.

[51] Charlson ME, Pompei P, Ales KL, MacKenzie CR (1987). A new method of classifying prognostic comorbidity in longitudinal studies: development and validation. Journal of Chronic Diseases.

[52] Elixhauser A, Steiner C, Harris DR, Coffey RM (1998). Comorbidity measures for use with administrative data. Medical Care.

[53] Puri R, Iung B, Cohen DJ, Rodés-Cabau J (2016). TAVI or No TAVI: identifying patients unlikely to benefit from transcatheter aortic valve implantation. European Heart Journal.

[54] Dreyfus J, Audureau E, Bohbot Y, Coisne A, Lavie-Badie Y, Bouchery M (2022). TRI-SCORE: a new risk score for in-hospital mortality prediction after isolated tricuspid valve surgery. European Heart Journal.

[55] Adamo M, Russo G, Pagnesi M, Pancaldi E, Alessandrini H, Andreas M (2024). Prediction of Mortality and Heart Failure Hospitalization After Transcatheter Tricuspid Valve Interventions: Validation of TRISCORE. JACC. Cardiovascular Interventions.

[56] LaPar DJ, Likosky DS, Zhang M, Theurer P, Fonner CE, Kern JA (2018). Development of a Risk Prediction Model and Clinical Risk Score for Isolated Tricuspid Valve Surgery. The Annals of Thoracic Surgery.

[57] Niro L, Delgado V (2024). Defining the Sweet Spot in Transcatheter Tricuspid Valve Interventions. JACC. Cardiovascular Interventions.

