# Supplementary material for: Heterotopic Caval Valve Implantation for Severe Tricuspid Regurgitation: A Systematic Review and Recommendations for Implantation and Futility
Source: Rev Cardiovasc Med. 2026 Jul 21;27(7):51423. doi: 10.31083/RCM51423 (PMC13419955; doi:10.31083/RCM51423)
Supplement: Supplementary file 1 [file 2153-8174-27-7-51423-s1.zip › Supplementary Material.docx]

**Heterotopic caval valve implantation for severe tricuspid regurgitation:**

**systematic review and recommendations for implantation and futility**

**Supplemental Appendix**

Supplementary Table 1. Registries included in this review. 2

Supplementary Table 2. Case series included in this review. 3

Supplementary Table 3. Methodological quality assessment of included studies

using the Joanna Briggs Institute (JBI) Critical Appraisal Tools 5

Supplementary Table 4. Details of Registries included in this study 7

Supplementary Table 5. Details of Case series included in this study 9

Supplementary Table 6. Details of the ongoing Studies/Registries. 13

References for supplemental material 14

PRISMA checklist. 17

**Supplementary Table 1. Registries included in this review.**

| Author, year | Study type | Population | Intervention | Comparator | Outcomes |
| --- | --- | --- | --- | --- | --- |
| William W. O’Neil, 2020 [1] | Retrospective observational registry study | Severe symptomatic TR despite OMT, high surgical risk | CAVI, n = 24 | NA | 30 days mortality and overall mortality, procedural success and related complications, NYHA class improvement |
| Rodrigo Estevez Loureiro, 2022 [2] | Prospective, non-randomized, single-arm, multicenter observational study | Severe symptomatic TR despite OMT, high surgical risk | CAVI, n = 35 | NA | 6 months mortality, readmission for HF, procedural success and related complications, improvements of symptoms, NYHA class, QoL and 6MWT |
| Mirjam G Wild, 2022 [3] | Retrospective observational registry study | Severe symptomatic TR despite OMT, high surgical risk and unsuitable for other transcatheter treatments | CAVI, n = 21 | NA | 30 days and 1 year mortality, Procedural success and related complications, readmission for HF, improvements in NYHA class and symptoms |
| Sara Blasco-Turrión, 2024 [4] | Prospective, non-randomized, single-arm, multicenter observational study | Severe symptomatic TR despite OMT, high surgical risk | CAVI, n= 44 | NA | 6 months and 1 year mortality, Procedural success and related complications, readmission for HF, improvements of symptoms, NYHA class, QoL, 6MWT |
| Angel Sánchez-Recalde, 2025 [5] | Prospective, non-randomized, single-arm, multicenter observational study | Severe or more symptomatic TR despite OMT, high surgical risk, unsuitable for orthotropic repair | CAVI, n = 204 | NA | Mortality, procedural success and related complications, improvements in NYHA class, symptoms, RV function and dimensions and major adverse events |

Included and selected registries according to PICO framework. CAVI, caval valve implantation; HF, heart failure; NA, not applicable; NYHA, New York Heart Association; OMT, optimized medical therapy; QoL, quality of life; RV, right ventricle; TR, tricuspid regurgitation; 6MWT, 6-minutes walking test.

**Supplementary Table 2. Case Series included in this review.**

| Author, year | Study type | Population | Intervention | Comparator | Outcomes |
| --- | --- | --- | --- | --- | --- |
| Laule Michael, 2013 [6] | Prospective non-randomized case series | Severe symptomatic TR despite OMT, high surgical risk | CAVI, n = 3 | NA | 30 days mortality, procedural related complications, NYHA class improvement |
| Lauten Alexander, 2018 [7] | Multicenter, Observational, early clinical experience | Severe symptomatic TR despite OMT, high surgical risk | CAVI, n = 25 | NA | 30 days and 12 months mortality, procedural success and related complications, NYHA class improvement |
| Wilbring Manuel, 2020 [8] | Single center observational case series | Severe symptomatic TR despite OMT, high surgical risk | CAVI, n = 2 | NA | Procedural success, readmission for HF |
| Sharkey Aidan, 2020 [9] | Retrospective observational case series | Severe symptomatic TR despite OMT, high surgical risk | CAVI (only in IVC), n= 2 | NA | Procedural success, mortality, improvement of renal function and resolution of abdominal pain |
| Aparisi Alvaro, 2020 [10] | Retrospective observational case series | Massive TR, high surgical risk | CAVI, n = 2 | NA | 30 days mortality, procedural success, improvements in NYHA class and 6MWT |
| Dreger Henryk, 2020 [11] | Randomized controlled trial | Severe symptomatic TR despite OMT, high surgical risk | CAVI (only in IVC), n = 14  OMT n = 14 | OMT and CAVI at 1,3,6 an 12 months | All cause mortality, readmission for HF, procedural related complications, NYHA class, 6-MWT and QoL improvement |
| Aalaei-Andabili Seyed Hossein, 2020 [12] | Retrospective observational case series | Severe symptomatic TR despite OMT, high surgical risk | CAVI (only in IVC), n = 6 | NA | Mortality, procedural success, readmission for HF, improvements of symptoms, TR and EF |
| Cruz-Gonza Ignacio, 2021 [13] | Prospective, multicenter and observational study | Severe symptomatic TR despite OMT, high surgical risk | CAVI, n = 6 | NA | Mortality, procedural success, readmission for HF, improvements in NYHA class, TR and RV function |
| Wild G Mirjam, 2021 [14] | Retrospective observational case series | Severe symptomatic TR despite OMT, high surgical risk | CAVI, n = 3 | NA | Mortality, procedural success, readmission for HF, improvements of symptoms, NYHA class and RV function, reduction of RV dimensions |
| Grazina Andrè, 2023 [15] | Retrospective observational case series | Severe and massive symptomatic TR despite OMT, high surgical risk | CAVI, n = 2 | NA | 6 months mortality, procedural success, readmission for heart failure, improvement in NYHA class |
| Di Mauro Michele, 2024 [16] | Retrospective, single center observational case series | Severe or more symptomatic TR despite OMT, high surgical risk | CAVI, n = 13 | NA | Mortality, survival rate, procedural success, readmission for HF, improvements of symptoms, NYHA class, TR, EF, RV function and dimensions |
| O’Neil P Brian, 2025 [17] | Prospective feasibility observational study | Severe symptomatic TR despite OMT, high surgical risk | CAVI (only in IVC), n = 10 | NA | 1 year mortality, procedural success and related complications, improvement in NYHA class, RV function, cardiac output and cardiac index |
| Bozbas Huseyin, 2025 [18] | Retrospective observational case series | Torrential TR not suitable for surgery or T-TEER | CAVI, n = 7 | NA | Mortality, procedural success improvements in NYHA class and symptoms |
| Lurz Philipp, 2025 [19] | Prospective single-arm, multicenter study | Severe symptomatic TR despite OMT, high surgical risk | CAVI, n = 20 | NA | 30 days mortality, procedural success and related complications, readmission for HF, improvements in NYHA class, QoL, 6-MWT, TR, RV and LV function and dimensions |

Included and selected Case series according to PICO framework. CAVI, caval valve implantation; EF, ejection fraction; HF, heart failure; IVC, inferior vena cava; LV, left ventricle; NA, not applicable; NYHA, New York Heart Association; OMT, optimized medical therapy; QoL, quality of life; RV, right ventricle; TR, tricuspid regurgitation; 6MWT, 6-minutes walking test.

**Supplementary Table 3. Methodological quality assessment of included studies using the Joanna Briggs Institute (JBI) Critical Appraisal Tools.**

| Author, year | Study type | JBI Tool | Q1 | Q2 | Q3 | Q4 | Q5 | Q6 | Q7 | Q8 | Q9 | Q10 | Q11 | Overall appraisal | Quality rating |
| --- | --- | --- | --- | --- | --- | --- | --- | --- | --- | --- | --- | --- | --- | --- | --- |
| William W. O’Neil, 2020 [1] | Registry | Cohort | Yes | Yes | Unclear | Yes | No | Yes | Unclear | Yes | Yes | Unclear | Yes | Moderate | Moderate |
| Rodrigo Estevez Loureiro, 2022 [2] | Prospective registry | Cohort | Yes | Yes | Yes | Yes | Unclear | Yes | Yes | Yes | Yes | Yes | Yes | Low | High |
| Mirjam G Wild, 2022 [3] | Multicenter registry | Cohort | Yes | Yes | Yes | Yes | Unclear | Yes | Yes | Yes | Yes | Yes | Yes | Low | High |
| Sara Blasco-Turrión, 2024 [4] | Cohort | Cohort | Yes | Yes | Yes | Yes | Yes | Yes | Yes | Yes | Yes | Yes | Yes | Low | High |
| Angel Sánchez-Recalde, 2025 [5] | Multicenter registry | Cohort | Yes | Yes | Yes | Yes | Unclear | Yes | Yes | Yes | Yes | Yes | Yes | Low | High |
| Laule Michael, 2013 [6] | First-in-man | Cohort | Yes | Yes | Unclear | Yes | Unclear | Yes | Unclear | Yes | No | Unclear | NA | Moderate | Moderate |
| Lauten Alexander, 2018 [7] | Case series | Case series | Yes | Yes | Yes | Yes | Unclear | Yes | Yes | Yes | Unclear | Yes | NA | Moderate | Moderate |
| Wilbring Manuel, 2020 [8] | Case series (n=2) | Case report | Yes | Yes | Yes | Yes | Unclear | Yes | Yes | Yes | NA | NA | NA | Moderate | Low |
| Sharkey Aidan, 2020 [9] | Case series | Case series | Yes | Yes | No | Yes | Unclear | Yes | No | Yes | No | Unclear | NA | High | Low |
| Aparisi Alvaro, 2020 [10] | Case series | Case series | Yes | Yes | Unclear | Yes | Unclear | Yes | Unclear | Yes | No | Unclear | NA | High | Low |
| Dreger Henryk, 2020 [11] | RCT | RCT | Yes | Yes | Yes | Yes | Yes | Yes | Yes | Yes | Yes | Yes | Yes | Moderate | High |
| Aalaei-Andabili Seyed Hossein, 2020 [12] | Case series | Case series | Yes | Yes | Unclear | Yes | Unclear | Yes | Unclear | Yes | No | Unclear | NA | High | Low |
| Cruz-Gonza Ignacio, 2021 [13] | Case series | Case series | Yes | Yes | Yes | Yes | Unclear | Yes | Yes | Yes | Unclear | Yes | NA | Moderate | Moderate |
| Wild G Mirjam, 2021 [14] | Case series (n=3) | Case report | Yes | Yes | Yes | Yes | Unclear | Yes | Yes | Yes | NA | NA | NA | Moderate | Low |
| Grazina Andrè, 2023 [15] | Case series (n=2) | Case report | Yes | Yes | Yes | Yes | Unclear | Yes | Yes | Yes | NA | NA | NA | Moderate | Low |
| Di Mauro Michele, 2024 [16] | Single-center cohort | Cohort | Yes | Yes | Unclear | Yes | Unclear | Yes | Yes | Yes | Yes | Unclear | Yes | Moderate | Moderate |
| O’Neil P Brian, 2025 [17] | Early feasibility cohort | Cohort | Yes | Yes | Yes | Yes | Unclear | Yes | Yes | Yes | Unclear | Yes | NA | Moderate | Moderate |
| Bozbas Huseyin, 2025 [18] | Case series | Case series | Yes | Yes | Unclear | Yes | Unclear | Yes | Unclear | Yes | No | Unclear | NA | High | Low |
| Lurz Philipp, 2025 [19] | Early feasibility cohort | Cohort | Yes | Yes | Yes | Yes | Unclear | Yes | Yes | Yes | Unclear | Yes | NA | Moderate | Moderate |

**Q1–Q8:** Items from the JBI Critical Appraisal Checklists, applied according to study design. **Overall appraisal** was derived from the proportion of “Yes” responses and used for descriptive purposes only. “Unclear” indicates insufficient reporting. NA, not applicable; JBI, Joanna Briggs Institute. **Quality rating** provides an overall assessment of methodological robustness for each study, integrating the study design, sample size, completeness of follow-up, generalizability, and risk of bias as assessed with the Joanna Briggs Institute (JBI) tools. Ratings are defined as follows: **High:** well-designed study with low risk of bias, adequate sample size, and complete follow-up; **Moderate:** study with some methodological limitations, unclear reporting, or partial follow-up; **Low:** small study, incomplete reporting, or high risk of bias, limiting confidence in the findings.

Check list for Case series: Munn Z, Barker TH, Moola S, Tufanaru C, Stern C, McArthur A, Stephenson M, Aromataris E. Methodological quality of case series studies: an introduction to the JBI critical appraisal tool. JBI Evidence Synthesis. 2020;18(10):2127-2133

For more information, visit: https://jbi.global/critical-appraisal-tools

**Supplementary Table 4. Details of Registries included in this study.**

| Author, year | N patients | Inclusion Criteria | Exclusion Criteria | Type of Valve | Outcomes | Procedural characteristics | Mortality | Adverse events |
| --- | --- | --- | --- | --- | --- | --- | --- | --- |
| William W. O’Neil, 2020 [1] | 24 (US Caval valve registry) | Severe symptomatic TR, high surgical risk and suitable for CAVI | IVC diameter > 29 mm Sapien 3 | Sapien 3 valve (Edwards) 29 mm (n=23),  23 mm (n=1) | At 30.5 days: NYHA class at least I 72.7%; only 1 patient (9.1%) considered worse at follow up.  No paravalvular leak 90% | PS: 100%. Bleeding 8.3%; MVC 4.2%; death procedure-related 4,17%; LOS 8.5 days | 30-days mortality 25%; In-hospital mortality 20.8%; overall mortality for 332 days: 58.3% | 1 death procedure related; high mortality < 1 year (58.3%); many deaths in the first 3 months after CAVI; Median survival: 350 days |
| Rodrigo Estevez Loureiro, 2022 [2] | 35 (TRICUS EURO STUDY) | Severe symptomatic TR despite OMT; LVEF ≧40%; 6MWT ≧ 60 m; ineligible for open heart surgery, clinical and anatomical suitable for CAVI | TAPSE < 13 mm and/or PASP > 65 mmHg, creatinine > 3 mg/dL or dyalisis within the past 4 weeks or at time of screening | TricValve (IVC+SVC) | At 6 months: KCCQ from 42.01 to 59.7; 79.4% patients in NYHA class I or II; HF 20%; Complete resolution (58.6%) or partial resolution (72.3%) of fluid overload; absence of hepatic vein backflow 52.9%. Reduction in the dose of loop diuretics | TS 97%; PS 94%; transitory shoulder pain 28.5%; device embolization/migration 3%; new PMK implantation 3%; major bleeding 17.1%; LOS 7 days | In-Hospital mortality 2.8%, 6-months mortality 8.5%. Death related to bleeding | Readmission of 7 patients (20%) for right HF and/or worsening of renal function. Increase of NT-proBNP due to ventricularization of RA. 6MWT improvement not statistically significant. No change in echocardiography |
| Mirjam G Wild, 2022 [3] | 21 | Severe symptomatic TR ineligible for surgery or other transcatheter treatment, clinical and anatomical suitability for TRICENTO | Not clinical and anatomical suitable for TRICENTO | TRICENTO (bicaval valved stent graft) | After 107 days: improvement of symptoms, NYHA class I or II. Congestion resolved in 37% of patients, still showing peripheral edema. Persisting ascites 5%. Reduced diuretics dose. Decrease of RV end diastolic volume in cardiac MRI | TS 100%; paraprosthetic leakage 9,52% (1 case severe); vascular complications 19%; MVC 5%; AKI 19% with 4.76% requiring dialysis; SIRS requiring IV vasopressors 4.76%; PMK 0%; LOS: 10 days | 30-days mortality: 5%, 1-year mortality: 24%. No in-hospital death | Death from cardiovascular causes 10%; readmission for right HF 19%; stent fracture 14% (unchanged valve function).  Echocardiography unchanged, peripheral edema unsolved. |
| Sara Blasco-Turrión, 2024 [4] | 44 (TRICUS + TRICUS EURO STUDY | Severe symptomatic TR despite OMT, high surgical risk, suitable for TricValve according to anatomic criteria. | TAPSE ≦13 mm, PASP > 65 mmHg, life expectancy < 1 year | TricValve (IVC+SVC) | At 1 year: KCCQ 56.4%; NYHA class I or class II 62.2%; 6MWT ≧ 40 m: 40%; Major bleeding due to AVK 20.4%; Stroke 9%; Right heart trombi 13.6%; TV surgery after 6 months: 2.3% due to SVC prothesis embolization. Decreased tricuspid annular diameter, no hepatic vein backflow. Lower diuretics doses. Lower NTpro-BNP | Paravalvular leak after 6 months: 6.8%, only 1 requiring percutaneous closure of the leak. | 6-months mortality: 6,8%; 1-year mortality: 0%; overall mortality: 6.8%; Cardiovascular death 2.2% | HF rehospitalization 29.5%, after 6 months: 20,45%, after 1 year: 9,09%. Death for cardiovascular causes 2,27% |
| Angel Sánchez-Recalde, 2025 [5] | 204 (TricBicaval Registry) | Symptomatic severe, massive or torrential TR with high surgical risk, unsuitable for tricuspid orthotopic repair | TAPSE < 13 mm, LVEF < 40%, PASP > 65 mmHg, caval V waves < 15 mmHg at RH catheterization | TricValve (IVC+SVC) | At 1 year: NYHA class I or class II 81.5%; peripheral edema 22.1%; ascites 4.9%; TAPSE and RV FAC worsened slightly but significantly; Hepatic vein systolic flow reversal decreased 26.2%. No changes in RA size, reduction in RV basal and mid ventricular end-diastolic diameter (non-significant) | PS 96.1%; clinical success at 30 days 83%; ViV 2.94%; bleeding 18.6%; shoulder pain 47.1%; vocal cord paralysis 1,47%; diaphragmatic paralysis 0,49%; new PMK implantation 33.3% (among cardiac complications); life threatening bleeding 3.9%; MVC 5.4%; AKI requiring dialysis 4.4%; device dysfunction requiring intervention 2%. LOS: 8 days |  | Readmission for HF 26.9%. In hospital mortality for cardiovascular causes: 76.5%; Cardiac complications 4.9%; Acute RV dysfunction treated with inotropes 7.8%; MAEs 19.1%; MCC 5.9%. |

AVK, antagonist vitamin K; AKI, acute kidney injury; CAVI, Caval valve implantation; HF, heart failure; KCCQ, Kansas City Cardiomyopathy Questionnaire; HF, heart failure; IVC, inferior vena cava; LOS, length of stay; LVEF, left ventricle ejection fraction; MAE, major adverse events; MCC, major cardiac complications; MVC, Major vascular complications; NYHA, New York Heart Association; PASP, pulmonary artery systolic pressure; PMK, pacemaker; PS, procedural success; RA, right atrium; RH, Right heart; RV, Right ventricle; SIRS, systemic inflammatory response syndrome; SVC, superior vena cava; TAPSE, tricuspid annular plane systolic excursion; TR, tricuspid regurgitation; TS, technical success; ViV, Valve-in-Valve.

**Supplementary Table 5. Details of Case series included in this study.**

| Author, year | N patients | Inclusion Criteria | Exclusion Criteria | Type of Valve | Outcomes | Procedural characteristics | Mortality | Adverse events |
| --- | --- | --- | --- | --- | --- | --- | --- | --- |
| Laule Michael, 2013 [6] | 3 | Severe symptomatic TR despite OMT, high surgical risk | NA | Edwards Sapien XT 29 mm (2 patients in IVC, 1 patient in IVC and SVC) | 30 days: improvement of at least 1 NYHA class; improvement of TAPSE), reduction of hepatic veins diameter, reduction of RA volume | No peri-procedural or in-hospital complications. No valve regurgitation or leakage | 30-days mortality 0% | 1 death procedure related; high mortality < 1 year (58.3%); many deaths in the first 3 months after CAVI; Median survival: 350 days |
| Lauten Alexander, 2018 [7] | 25 | Severe symptomatic TR despite OMT, high surgical risk (compassionate clinical use) | PASP > 60 mmHg, TAPSE < 10 mm, life expectancy < 3 months | Edwards Sapien XT or Sapien 3 (n=17), Tricvalve (n=7) Directflow (n=1). IVC only (n=19), BiCAVI (n=6) | 316 days in patients discharged (n=19): NYHA improvement 84.2%; NYHA class I or II 50.2% | PS 92%; valve migration 8%; device embolization 8% | 30 days mortality 12%; In-Hospital mortality 24%; 1-year mortality 63% | Patients dying from non cardiovascular causes despite successful treatment of TR. STS 14 |
| Wilbring Manuel, 2020 [8] | 2 | NA | NA | TRICENTO (bicaval valved stent graft) | First 3 months: recurrent signs of HF. Reduced RV auto regulation -> Recurrent episodes of HF | PS 100%. Nearly complete systolic compression of the stent graft at the level of the right atrium -> systemic back flow. | - | Persisting TR -> functional ventricularization of RA. |
| Sharkey Aidan, 2020 [9] | 2 | Severe symptomatic TR despite OMT, high surgical risk | NA | Edwards Sapien 3 in IVC | Improved renal function and resolution of abdominal pain | PS 100% | **Case 1:** 100% after 11 months.  **Case 2:** 100% after 9 months | **Case 1:** death for cardiac arrest, then cardiogenic shock and sepsis.  **Case 2:** aspiration pneumonia led to death |
| Aparisi Alvaro, 2020 [10] | 2 | **Case 1:** massive TR, preserved RV function.  **Case 2:** massive TR, decompensated right HF | NA | **Case 1:** Tricento.  **Case 2:** Tricvalve. | **Case 1:** at 30 days, improvement in NYHA class, 6-MWT and reduction of TR.  **Case 2:** at 30 days, improvement in NYHA class and 6-MWT. Persisting TR as severe, reduction of PAPs (from 68 to 49 mmHg) | PS 100% | 30-days mortality: 0% |  |
| Dreger Henryk, 2020 [11] | CAVI n=14  OMT  [20]n= 14 | Severe symptomatic TR despite OMT, high surgical risk | LVEF < 30%, regular dialysis or serum creatinine > 3 mg/dl, life expectancy < 12 months, IVC diameter > 31 mm | Edwards Sapien XT 23, 26 or 29 mm in IVC | Comparing OMT and CAVI at 1,3,6 and 12-months: no differences in maximal oxygen uptake measured by spiroergometry, NYHA class improved by 1 class (46% OMT vs 63% CAVI) in 3 months with no differences over the entire follow up. QoL (MLHFQ), 6-MWT, NT-proBNP and RH function: no differences. | 4 conversion (28.57%) to open heart surgery for valve dislocation and stent migration -> stop recruitment | 1-year mortality: OMT 29%, CAVI 57%. RH failure: OMT 21%, CAVI 4% | Heart failure hospitalization: OMT 29%, CAVI 29% |
| Aalaei-Andabili Seyed Hossein, 2020 [12] | 6 | Severe symptomatic TR despite OMT, high surgical risk | NA | Edwards Sapien 3 in IVC | 30 days: improvements of symptoms (ascites, peripheral edema and physical capacity), improvement of TR and EF (except in 2 patients) | PS 100% | 16,67% (n=1) died after 3 months with pre-procedural severe RV disfunction | 66,67% (n=4) readmitted within 6 months for shortness of breath, lower extremity edema and ascites |
| Cruz-Gonza Ignacio, 2021 [13] | 6 | Severe symptomatic TR despite OMT, high surgical risk | TAPSE ≦13 mm,  LVEF ≦ 30%, PASP > 70 mmHg or unfavorable anatomy by MSCT | Tricento | 11 months: NYHA improvement (I-II). 6-months follow up: reduction of TR grade, vena contact width, tricuspid angular diameter and RA volume. Improvement of RV function but not statistically significant. Reduction of diuretics doses. NT-proBNP decrease not statistically significant. | PS 100% | 11 months: 0% | One patient with acute decompensation of HF (41 days after the procedure); one patient with early discharge follow-up for therapeutical optimization |
| Wild G Mirjam, 2021 [14] | 3 | Severe symptomatic TR despite OMT, high surgical risk, unsuitable for other treatment options | NA | Novel custom-made bicaval valved stent (Medira AG) | Symptomatic improvement (NYHA class, resolution of ascites, peripheral edema, pleural effusion and ascites). Reduction of RV dimensions. | PS 100% | **Case 1:** 2 years -> alive;  **Case 2:** 21 months -> dead;  **Case 3:** 21 months -> alive  Overall mortality: 33,3% | **Case 1:** systolic compression of stent in RA without an impact on valve competency.  **Case 2:** dead by terminal RV failure.  **Case 3:** increased systolic compression of stent from tangential impact of the regurgitant jet of TR in the RA (suspected stent fracture) |
| Grazina Andrè, 2023 [15] | 2 | Severe and massive symptomatic TR despite OMT, high surgical risk, unsuitable for other treatment options | NA | Tricvalve | After 6 months: NYHA class II, no other hospital admissions, clinically well. | **Case 1:** procedural success 100%. After 3 months: valve in valve.  **Case 2:** migration of SVC prothesis in RA -> ViV | 6 months: 0 | **Case 1:** 3 moths after, perivalvular leak in IVC with flow inversion in supra-hepatic veins -> ViV  **Case 2**: ViV in the same procedure for migration of SVC prosthesis in RA. |
| Di Mauro Michele, 2024 [16] | 13 | Severe or more symptomatic TR despite OMT, high surgical risk, suitable for tricvalve,  LVEF ≧35% | PASP > 65 mmHg, life expectancy < 1 year | Tricvalve | After 170 days: No signs of RHF. Improved NYHA class in 82% (NYHA II in 73%, NYHA I in 9%). Increase of LVEF, reduction of RV diameter and dysfunction. Improvement in TR grade due to right ventricle remodeling and reducing annular dimension/tethering, decrease of NT-proBNP and diuretics dose. No hospitalization. No ascites or peripheral edema | PS 100% | 15,4% (n=2) patients died.  After 170 days, survival rate of 80.2% | 2 patients with triscore 65% were operated and then died due to renal or liver failure (6 days and 124 days after the procedure, respectively) |
| O’Neil P Brian, 2025 [17] | 10 | Severe symptomatic TR despite OMT, high surgical risk | LVEF ≦ 20%, PASP > 70 mmHg , severe RV dysfunction, severe renal impairment or chronic renal replacement therapy | Edwards Sapien 3 in IVC (26 mm) + Edwards Caval prestent | After 6 months: RA, SVC and IVC pressure decreased, CO and CI increased.  At 30 days: KCCQ-OS score improved.  At 1 year: NYHA class improvement (50% in NYHA class I and 50% in NYHA class II). Improved RV dysfunction 12.5%. Mean hepatic vein dimension decreased. | PS 90%, 1 impossibility to measure IVC diameter (probably too large).  No major bleeding or vascular complications during the procedure | 1-year mortality: 20%. | Myocardial infarction at 1 year: 10%. Major bleeding at 1 year: 10%. 30% hospitalized for HF in the first year.  POD day 37 and 204 revealed pre-stent fracture in 70% patients |
| Bozbas Huseyin, 2025 [18] | 7 | Torrential TR not suitable for surgical intervention or T-TEER due to severe computation defects | NA | Tricvalve | **Case 1,4,6:** discharged with NYHA class II. Decreased doses of diuretics and peripheral edema.  **Case 2:** died a week after the procedure due to MOF.  **Case 3:** decrease of diuretics doses.  **Case 5, 7:** no outcomes available. | PS 100% | 14,29% (n=1) death due to MOF after 1 week | 1 death one week after the procedure (14,29%) |
| Lurz Philipp, 2025 [19] | 20 | Severe symptomatic TR despite OMT, high surgical risk, suitable for cross-caval stent graft | LVEF < 20%, TAPSE < 13 mm, unfavourable anatomy of SVC or IVC, PASP > 70 mmHg, renal (eGFR < 30 ml/min/1.73m2) and liver (MELD > 20) impairment, CFS ≧ 7 | Trillium Device | At 30 days: TR reduced to mild (100%), RV function and dimension not affected by treatment, as LV function and dimension. Improvement of NYHA class (50% patients in NYHA class I or II), reduction in the edema severity score (75% patients with score I or II). Increase in KCCQ score. Nonsignificant increase 6MWT. Reduction of bilirubin. Increase of NT-proBNP and lactate dehydrogenase | PS 100%. 1 case of mild paravalvular inferior vena cava leak. | 30 days mortality: 5% (n=1) due to progressive HF and MOF | 30-days complications: renal failure requiring hemodialysis 10% (n=2); major GI bleeding requiring intervention or blood transfusion 10% (n=2); Post-implantation syndrome with elevated leukocyte and C-reactive protein for an average of 4 days 25% (n=5) |

AVK, antagonist vitamin K; AKI, acute kidney injury; CAVI, Caval valve implantation; CFI, clinical frailty scale; CI, Cardiac Index; CO, Cardiac Output; eGFR, estimated glomerular filtration rate; HF, heart failure; KCCQ, Kansas City Cardiomyopathy Questionnaire; IVC, inferior vena cava; LOS, length of stay; LVEF, left ventricle ejection fraction; MAE, major adverse events; MCC, major cardiac complications; MELD, model for end stage liver disease; MOF, multi organ failure; MSCT, Multi-slice Computed Tomography; MVC, Major vascular complications; NA, Not Available; NYHA, New York Heart Association; PASP, pulmonary artery systolic pressure; PMK, pacemaker; PS, procedural success; RA, right atrium; RH, Right heart; RV, Right ventricle; SIRS, systemic inflammatory response syndrome; SVC, superior vena cava; TAPSE, tricuspid annular plane systolic excursion; TR, tricuspid regurgitation; TS, technical success; ViV, Valve-in-Valve.

**Supplementary Table 6. Details of the ongoing Studies/Registries.**

| Name of study/Registry | Year | NCT | Type of Valve | Design of the study | N of patients | Primary Outcomes | State |
| --- | --- | --- | --- | --- | --- | --- | --- |
| TRICAV-II Pivotal: TRIcvalve biCAVal ventilsystem for severe tricuspid regurgitation | 2024 | NCT06458907 | TricValve® | Perspective Trial / Pivotal | 600 | 1) Mortality; 2) MAE; 3) reduction of regurgitant flow in vena cava 4) improving of QoL (KCCQ) 5) HF events | Not yet recruiting (completion in 2030) |
| European Registry of Transcatheter Repair for Tricuspid Regurgitation (EuroTR) | 2023 | NCT06307262 | TricValve®, Evoque, LuX-Valve | European observational registry | 3000 | 1) Mortality | Recruiting (completion in 2030) |
| Retrospective Prospective Multicentric Clinical Follow-up of Patients After Being Treated With TricValve® | 2022 | NCT05114850 | TricValve® | Multicentred observation cohort | 450 | **1**) Number of patients re-admitted for HF | Recruiting (completion in 2028) |
| Innoventric Trillium Stent Graft First-in-Human (FIH) Study | 2020 | NCT04289870 | Innoventric Trillium™ Stent Graft System | Interventional, single arm, non randomized clinical study | 20 | 1) safety endpoint; 2) technical performance; 3) efficacy endpoint | Active, not recruiting (completion 2026) |
| TRICUS STUDY euro - safety and efficacy of the TricValve Device | 2019 | NCT04141137 | TricValve® | Observational European registry | 35 | 1) MAE; 2) improving of QoL (KCCQ) | Completed (in 2021) |
| TRICUS STUDY - Safety and Efficacy of the TricValve® Device | 2018 | NCT03723239 | TricValve® | Observational European registry | 9 | 1) MAE; 2) improving of NYHA | Completed (in 2021) |
| Treatment of Severe Secondary TRIcuspid Regurgitation in Patients With Advance Heart Failure With CAval Vein Implantation of the Edwards Sapien XT VALve (TRICAVAL) | 2015 | NCT02387697 | Edwards sapien XT valve (IVC) | Randomized clinical trials | 28 | 1) maximum relative VO2 uptake; 2)NYHA class; 3)EF; 4) RV, RA and hepatic vein diameter; 5) NT-proBNP; 6) TR jet VTI; 6) HF questionnaire; 7) 6MWT; 8) unscheduled rehospitalization; 9) dyspnoea VAS; 10) ventilation efficiency; 11) aerobic threshold | Completed (2018) |

EF, ejection fraction; HF, heart failure; KCCQ, Kansas City Cardiomyopathy Questionnaire; IVC, inferior vena cava; MAE, major adverse events; NYHA, New York Heart Association; RA, right atrium; RH, Right heart; RV, Right ventricle; TR, tricuspid regurgitation; VAS, visual analogue scale; VO2, volume of oxygen; VTI, velocity time integral; 6MWT, 6 minutes walking test

**References for supplemental material**

1. O'Neill B. P, Negrotto S, Yu D, et Al. Caval Valve Implantation for Tricuspid Regurgitation: Insights From the United States Caval Valve Registry. J Invasive Cardiol, 2020, pp. 470-475. DOI: 10.25270/jic/20.00371.

2. Estévez-Loureiro, R, Sánchez-Recalde, A, Amat-Santos, I. et al. 6-Month Outcomes of the TricValve System in Patients With Tricuspid Regurgitation: The TRICUS EURO Study. J Am Coll Cardiol Intv., 2022, pp. 1366-1377. https://doi.org/10.1016/j.jcin.2022.05.022.

3. Wild, M. G., Lubos, E., Cruz-Gonzalez, I., Amat-Santos, I., Ancona, M., Andreas, M., Boeder, N. F., Butter, C., Carrasco-Chinchilla, F., Estevez-Loureiro, R., Kempfert, J., Köll, B., Montorfano, M., Nef, H. M., Toggweiler, S., Unbehaun, A., et Al. Early Clinical Experience With the TRICENTO Bicaval Valved Stent for Treatment of Symptomatic Severe Tricuspid Regurgitation: A Multicenter Registry. Circulation. Cardiovascular interventions, 2022, Vol. 15(3), p. e011302. https://doi.org/10.1161/CIRCINTERVENTIONS.121.011302.

4. Blasco-Turrión, S, Briedis, K, Estévez-Loureiro, R. et al. Bicaval TricValve Implantation in Patients With Severe Symptomatic Tricuspid Regurgitation: 1-Year Follow-Up Outcomes. J Am Coll Cardiol Intv., 2024, Vol. 17(1), pp. 60-72. https://doi.org/10.1016/j.jcin.2023.10.043.

5. Angel Sánchez-Recalde, Luis M. Domínguez-Rodríguez, Liesbeth Rosseel, Luis Nombela-Franco, Roman Pfister, Ignacio Amat-Santos, Christian Butter, Michele Di Mauro, Ignacio Cruz-González, Xavier Freixa, Martin Swaans, Christoph Wilde, Duarte Cacela et Al. Bicaval TricValve Implantation in Patients With Severe Tricuspid Regurgitation: 1-Year Outcomes From the TricBicaval Registry. JACC: Cardiovascular Interventions, 2025, Vol. 18(15), pp. 1913-1924. https://doi.org/10.1016/j.jcin.2025.06.023..

6. Laule, M., Stangl, V., Sanad, W., Lembcke, A., Baumann, G., & Stangl, K. Percutaneous transfemoral management of severe secondary tricuspid regurgitation with Edwards Sapien XT bioprosthesis: first-in-man experience. Journal of the American College of Cardiology., 2013, Vol. 61(18), pp. 1929–1931. https://doi.org/10.1016/j.jacc.2013.01.070.

7. Lauten, A., Figulla, H. R., Unbehaun, A., Fam, N., Schofer, J., Doenst, T., Hausleiter, J., Franz, M., Jung, C., Dreger, H., Leistner, D., Alushi, B., Stundl, A., Landmesser, U., Falk, V., Stangl, K., & Laule, M. Interventional Treatment of Severe Tricuspid Regurgitation: Early Clinical Experience in a Multicenter, Observational, First-in-Man Study. Circulation. Cardiovascular interventions., 2018, Vol. 11(2), p. e006061. https://doi.org/10.1161/CIRCINTERVENTIONS.117.006061.

8. Wilbring, M., Tomala, J., Ulbrich, S., Murugaboopathy, V., Matschke, K., & Kappert, U. Recurrence of Right Heart Failure After Heterotopic Tricuspid Intervention: A Conceptual Misunderstanding?. JACC. Cardiovascular interventions., 2020, Vol. 13(10), pp. e95–e96. https://doi.org/10.1016/j.jcin.2020.02.012.

9. Sharkey, A., Munoz Acuna, R., Belani, K., Sharma, R. K., Chaudhary, O., Fatima, H., Laham, R., & Mahmood, F. Heterotopic caval valve implantation for the management of severe tricuspid regurgitation: a case series. European heart journal. Case reports, 2020, Vol. 5(1), p. ytaa428. https://doi.org/10.1093/ehjcr/ytaa428.

10. Aparisi, Á., Amat-Santos, I. J., Serrador, A., Rodríguez-Gabella, T., Arnold, R., & San Román, J. A. Current clinical outcomes of tricuspid regurgitation and initial experience with the TricValve system in Spain. Revista espanola de cardiologia, 2020, Vol. 73(10), pp. 853–854. https://doi.org/10.1016/j.rec.2020.03.001.

11. Dreger, H., Mattig, I., Hewing, B., Knebel, F., Lauten, A., Lembcke, A., Thoenes, M., Roehle, R., Stangl, V., Landmesser, U., Grubitzsch, H., Stangl, K., & Laule, M. Treatment of Severe TRIcuspid Regurgitation in Patients with Advanced Heart Failure with CAval Vein Implantation of the Edwards Sapien XT VALve (TRICAVAL): a randomised controlled trial. EuroIntervention : journal of EuroPCR in collaboration with the Working Group on Interventional Cardiology of the European Society of Cardiology, 2020, Vol. 15(17), pp. 1506–1513. . https://doi.org/10.4244/EIJ-D-19-00901.

12. Aalaei-Andabili, S. H., Bavry, A. A., Choi, C., Arnaoutakis, G., Anderson, R. D., & Beaver, T. M. Percutaneous Inferior Vena Cava Valve Implantation May Improve Tricuspid Valve Regurgitation and Cardiac Output: Lessons Learned. Innovations (Philadelphia, Pa.), 2020, Vol. 15(6), pp. 577–580. https://doi.org/10.1177/1556984520957144.

13. Cruz-González, I., González-Ferreiro, R., Amat-Santos, I. J., Carrasco-Chinchilla, F., Alonso Briales, J. H., & Estévez-Loureiro, R. TRICENTO transcatheter heart valve for severe tricuspid regurgitation. Initial experience and mid-term follow-up. Revista espanola de cardiologia, 2021, Vol. 74(4), pp. 351–354. https://doi.org/10.1016/j.rec.2020.09.016.

14. Wild, M. G., Gloeckler, M., Wustmann, K. B., Erne, S. A., Grogg, H., Huber, A. T., Windecker, S., Praz, F., & Gräni, C. Multimodality Imaging for Evaluation of Bicaval Valved Stent Implantation in Severe Tricuspid Regurgitation JACC. Case reports., 2021, Vol. 3(13), pp. 1512–1518. https://doi.org/10.1016/j.jaccas.2021.07.009.

15. Grazina, A., Ferreira, A., Ramos, R., & Cacela, D. Heterotopic caval valve-in-valve procedure for prosthetic migration: two case reports. European heart journal Case reports., 2023, Vol. 7(8), p. ytad368. https://doi.org/10.1093/ehjcr/ytad368.

16. Di Mauro, M., Guarracini, S., Mazzocchetti, L., Capuzzi, D., Salute, L., Di Marco, M., Lorusso, R., & Calafiore, A. M. Transcatheter bicaval valve system for the treatment of severe isolated tricuspid regurgitation. Features from a single-Centre experience. International journal of cardiology, 2024, https://doi.org/10.1016/j.ijcard.2024.131864.

17. O'Neill, B. P., Amoroso, N. S., Yadav, P., Houston, B. A., Villablanca, P., O'Neill, W. W., Wang, D. D., Thourani, V. H., & Tedford, R. J. Early Feasibility Study of the Edwards SAPIEN 3 Transcatheter Heart Valve System With the Edwards Caval Prestent for the Treatment of Reverse Caval Flow in Patients With Severe Tricuspid Regurgitation (TR). Catheterization and cardiovascular interventions : official journal of the Society for Cardiac Angiography & Interventions, 2025, Vol. 106(4), pp. 2273–2281. https://doi.org/10.1002/ccd.70071.

18. Bozbaş, H., Barçın, C., Asfour, M., Çelebi, S. A., Çam, E., & İlkay, E. Caval Valve Implantation Procedure in 7 Cases of Torrential Tricuspid Regurgitation and Step-by-Step Description of the Procedure. Anatolian journal of cardiology., 2025, Vol. 29(5), pp. 261–264. https://doi.org/10.14744/AnatolJCardiol.2025.4750.

19. Lurz, P., Kresoja, K. P., Besler, C., Verheye, S., Vermeersch, P., Rudolph, V., Friedrichs, K., Abdul-Jawad Altisent, O., Freixa, X., Sanchis, L., Cruz-Gonzalez, I., Antunez-Muiños, P., Bartunek, J., Vanderheyden, M., Sherif, M., Trippel, T. D., et Al. Heterotopic Crosscaval Transcatheter Tricuspid Valve Replacement for Patients With Tricuspid Regurgitation: The Trillium Device. JACC. Cardiovascular interventions, 2025, Vol. 18(11), pp. 1425–1434. <https://doi.org/10.1016/j.jcin.2025.04.036>.

**PRISMA checklist**

| **Section and Topic** | **Item #** | **Checklist item** | **Location where item is reported** |
| --- | --- | --- | --- |
| **TITLE** | | |  |
| Title | 1 | Identify the report as a systematic review. | 1 |
| **ABSTRACT** | | |  |
| Abstract | 2 | See the PRISMA 2020 for Abstracts checklist. | 2 |
| **INTRODUCTION** | | |  |
| Rationale | 3 | Describe the rationale for the review in the context of existing knowledge. | 1-2 |
| Objectives | 4 | Provide an explicit statement of the objective(s) or question(s) the review addresses. | 1-2 |
| **METHODS** | | |  |
| Eligibility criteria | 5 | Specify the inclusion and exclusion criteria for the review and how studies were grouped for the syntheses. | 2-3 |
| Information sources | 6 | Specify all databases, registers, websites, organisations, reference lists and other sources searched or consulted to identify studies. Specify the date when each source was last searched or consulted. | 2-3 |
| Search strategy | 7 | Present the full search strategies for all databases, registers and websites, including any filters and limits used. | 2-3 |
| Selection process | 8 | Specify the methods used to decide whether a study met the inclusion criteria of the review, including how many reviewers screened each record and each report retrieved, whether they worked independently, and if applicable, details of automation tools used in the process. | 2-3 |
| Data collection process | 9 | Specify the methods used to collect data from reports, including how many reviewers collected data from each report, whether they worked independently, any processes for obtaining or confirming data from study investigators, and if applicable, details of automation tools used in the process. | 2-3 |
| Data items | 10a | List and define all outcomes for which data were sought. Specify whether all results that were compatible with each outcome domain in each study were sought (e.g. for all measures, time points, analyses), and if not, the methods used to decide which results to collect. | 2-3 |
|  | 10b | List and define all other variables for which data were sought (e.g. participant and intervention characteristics, funding sources). Describe any assumptions made about any missing or unclear information. | 2-3 |
| Study risk of bias assessment | 11 | Specify the methods used to assess risk of bias in the included studies, including details of the tool(s) used, how many reviewers assessed each study and whether they worked independently, and if applicable, details of automation tools used in the process. | 2-3 + suppl |
| Effect measures | 12 | Specify for each outcome the effect measure(s) (e.g. risk ratio, mean difference) used in the synthesis or presentation of results. | 2-3 + suppl |
| Synthesis methods | 13a | Describe the processes used to decide which studies were eligible for each synthesis (e.g. tabulating the study intervention characteristics and comparing against the planned groups for each synthesis (item #5)). | 2-3 |
|  | 13b | Describe any methods required to prepare the data for presentation or synthesis, such as handling of missing summary statistics, or data conversions. | 2-3 |
|  | 13c | Describe any methods used to tabulate or visually display results of individual studies and syntheses. | 2-3 |
|  | 13d | Describe any methods used to synthesize results and provide a rationale for the choice(s). If meta-analysis was performed, describe the model(s), method(s) to identify the presence and extent of statistical heterogeneity, and software package(s) used. | 2-3 |
|  | 13e | Describe any methods used to explore possible causes of heterogeneity among study results (e.g. subgroup analysis, meta-regression). | 2-3 |
|  | 13f | Describe any sensitivity analyses conducted to assess robustness of the synthesized results. | 2-3 |
| Reporting bias assessment | 14 | Describe any methods used to assess risk of bias due to missing results in a synthesis (arising from reporting biases). | 2-3 + Suppl |
| Certainty assessment | 15 | Describe any methods used to assess certainty (or confidence) in the body of evidence for an outcome. | 2-3 |
| **RESULTS** | | |  |
| Study selection | 16a | Describe the results of the search and selection process, from the number of records identified in the search to the number of studies included in the review, ideally using a flow diagram. | 3-6 |
|  | 16b | Cite studies that might appear to meet the inclusion criteria, but which were excluded, and explain why they were excluded. | 5 |
| Study characteristics | 17 | Cite each included study and present its characteristics. | 3-6 + suppl |
| Risk of bias in studies | 18 | Present assessments of risk of bias for each included study. | 3-6 + suppl |
| Results of individual studies | 19 | For all outcomes, present, for each study: (a) summary statistics for each group (where appropriate) and (b) an effect estimate and its precision (e.g. confidence/credible interval), ideally using structured tables or plots. | 3-6 + suppl |
| Results of syntheses | 20a | For each synthesis, briefly summarise the characteristics and risk of bias among contributing studies. | 3-6 + suppl |
|  | 20b | Present results of all statistical syntheses conducted. If meta-analysis was done, present for each the summary estimate and its precision (e.g. confidence/credible interval) and measures of statistical heterogeneity. If comparing groups, describe the direction of the effect. | NA |
|  | 20c | Present results of all investigations of possible causes of heterogeneity among study results. | 3-6 + suppl |
|  | 20d | Present results of all sensitivity analyses conducted to assess the robustness of the synthesized results. | NA |
| Reporting biases | 21 | Present assessments of risk of bias due to missing results (arising from reporting biases) for each synthesis assessed. | NA |
| Certainty of evidence | 22 | Present assessments of certainty (or confidence) in the body of evidence for each outcome assessed. | NA |
| **DISCUSSION** | | |  |
| Discussion | 23a | Provide a general interpretation of the results in the context of other evidence. | 6-13 |
|  | 23b | Discuss any limitations of the evidence included in the review. | 13 |
|  | 23c | Discuss any limitations of the review processes used. | 13 |
|  | 23d | Discuss implications of the results for practice, policy, and future research. | 13 |
| **OTHER INFORMATION** | | |  |
| Registration and protocol | 24a | Provide registration information for the review, including register name and registration number, or state that the review was not registered. | NA |
|  | 24b | Indicate where the review protocol can be accessed, or state that a protocol was not prepared. | NA |
|  | 24c | Describe and explain any amendments to information provided at registration or in the protocol. | NA |
| Support | 25 | Describe sources of financial or non-financial support for the review, and the role of the funders or sponsors in the review. | 14 |
| Competing interests | 26 | Declare any competing interests of review authors. | 14 |
| Availability of data, code and other materials | 27 | Report which of the following are publicly available and where they can be found: template data collection forms; data extracted from included studies; data used for all analyses; analytic code; any other materials used in the review. | NA |

*From:*  Page MJ, McKenzie JE, Bossuyt PM, Boutron I, Hoffmann TC, Mulrow CD, et al. The PRISMA 2020 statement: an updated guideline for reporting systematic reviews. BMJ 2021;372:n71. doi: 10.1136/bmj.n71. This work is licensed under CC BY 4.0. To view a copy of this license, visit <https://creativecommons.org/licenses/by/4.0/>

For more information, visit: <http://www.prisma-statement.org/>
